# Genome-scale network model of metabolism and histone acetylation reveals metabolic dependencies of histone deacetylase inhibitors

**DOI:** 10.1186/s13059-019-1661-z

**Published:** 2019-03-01

**Authors:** Fangzhou Shen, Luigi Boccuto, Rini Pauly, Sujata Srikanth, Sriram Chandrasekaran

**Affiliations:** 1 Department of Biomedical Engineering, University of Michigan, Ann Arbor, MI 48109 USA; 2 Greenwood Genetics Center, Greenwood, SC 29646 USA; 3 Center for Computational Medicine and Bioinformatics, University of Michigan, Ann Arbor, MI 48109 USA; 4 Rogel Cancer Center, University of Michigan, Ann Arbor, MI 48109 USA

**Keywords:** Genome-scale metabolic modeling, Metabolic network, Protein acetylation, Metabolic regulation, Protein deacetylases (KDACs), KDAC inhibitors

## Abstract

Histone acetylation plays a central role in gene regulation and is sensitive to the levels of metabolic intermediates. However, predicting the impact of metabolic alterations on acetylation in pathological conditions is a significant challenge. Here, we present a genome-scale network model that predicts the impact of nutritional environment and genetic alterations on histone acetylation. It identifies cell types that are sensitive to histone deacetylase inhibitors based on their metabolic state, and we validate metabolites that alter drug sensitivity. Our model provides a mechanistic framework for predicting how metabolic perturbations contribute to epigenetic changes and sensitivity to deacetylase inhibitors.

**Electronic supplementary material:**

The online version of this article (10.1186/s13059-019-1661-z) contains supplementary material, which is available to authorized users.

## Background

Protein acetylation is a highly conserved mechanism to regulate many cellular processes including transcription and metabolism. The substrate for acetylation, acetyl-CoA, is a central metabolic intermediate at the crossroads of anabolic and catabolic pathways [1]. Protein acetylation is thus sensitive to the metabolic state of the cell [2]. Biochemical assays in *Saccharomyces cerevisiae* and human cell lines have shown that levels of acetyl-CoA directly affect protein acetylation [3–5]. Hundreds of proteins, including metabolic enzymes, are regulated by acetylation [6, 7]. Acetylation can also influence gene expression through post-translational modification of histones. Cells rely on histone acetylation to increase chromatin accessibility and influence gene expression [2, 8].

Given its pervasive regulatory role, altered acetylation is believed to play a part in a variety of diseases including cancer and metabolic disorders such as diabetes, obesity, dyslipidemia, and hypertension [5, 9–11]. Since metabolic alterations and dysregulation of protein acetylation are important cancer hallmarks, understanding the interplay between these processes can reveal novel therapeutic targets against cancer. However, predicting the interplay between these two processes is challenging due to acetyl-CoA’s pervasive role in metabolism, and due to the highly interconnected nature of the metabolic network. No theoretical approach exists to predict the impact of the change in cellular metabolism on protein acetylation.

Creating a model of metabolism and protein acetylation can enable the prediction of the impact of nutrient shifts or mutations in metabolic enzymes on the epigenome. This can shed light on metabolic and chromatin dysregulation during tumorigenesis [12, 13]. Compounds that disrupt acetylation machinery such as deacetylase inhibitors are increasingly used for treating cancers and metabolic and immune disorders [10]. Predicting the interplay between metabolism and acetylation can identify cancer cells that are sensitive to deacetylase inhibitors based on their metabolic state.

To address this challenge, here we develop a computational model of metabolism and protein acetylation using constraint-based modeling (CBM). CBM makes use of metabolic network reconstructions that represent the mechanistic relationships between genes, proteins, and metabolites within a biological system. CBM has been successfully used to predict the metabolic state of various mammalian systems, including cancer cells and stem cells [14–17]. We hypothesized that protein acetylation dynamics can be inferred from the metabolic network topology and stoichiometry.

We demonstrate that our metabolic model can explain known acetylation changes associated with nutrient excess and starvation based on the availability of carbon units. We then apply our acetylation model to predict and validate the impact of cellular metabolic state on sensitivity to drugs that disrupt acetylation, specifically protein deacetylase inhibitors that are currently used in the clinic for anticancer therapy. Our approach allowed us to predict the variation in sensitivity between deacetylase inhibitors based on their unique impact on cellular metabolism.

## Results

### Simulating the effect of the metabolic state on acetylation

To simulate the influence of metabolism on acetylation, a nuclear protein acetylation reaction (protein + acetyl-CoA → acetyl-protein + CoA) was incorporated into the human metabolic network reconstruction by Duarte et al., which contains 3747 reactions, 1496 ORFs, 2004 proteins, and 2766 metabolites [18]. A nuclear ATP citrate lyase reaction and nuclear transport of citrate and oxaloacetate were also included to enable synthesis of acetyl-CoA in the nucleus based on recent biochemical evidence [19]. Since acetyl-CoA and substrates for acetyl-CoA synthesis can diffuse between the cytosol and nucleus through the nuclear pore, the flux through the protein acetylation reaction is representative of acetylation changes in both cytosol and nuclear proteins.

**Fig. 1:**
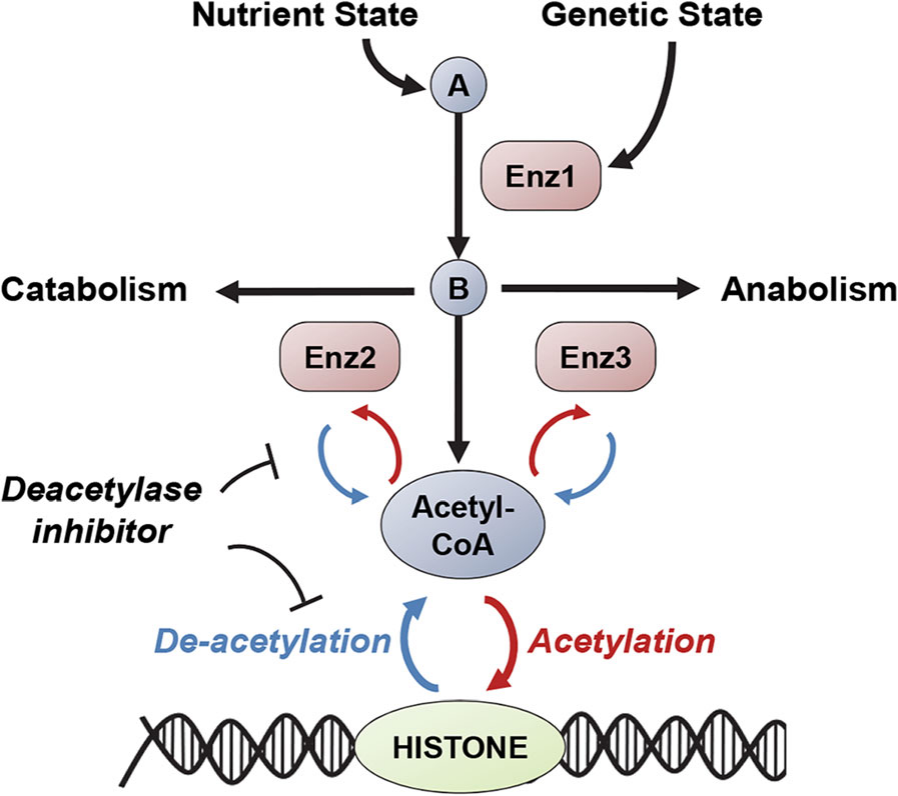
Overview of our systems approach to predict the impact of metabolism on acetylation. Using a genome-scale metabolic model, we first simulate the effect of different nutrient environments and gene deletions on protein acetylation. We then simulate the impact of the cellular metabolic state on vulnerability to compounds that disrupt acetylation (protein deacetylase inhibitors). We subsequently predict variation in susceptibility to these drugs between different cell types depending on their metabolic state. Abbreviations—A, B are metabolites and Enz1, Enz2, and Enz3 are metabolic enzymes

**Fig. 2:**
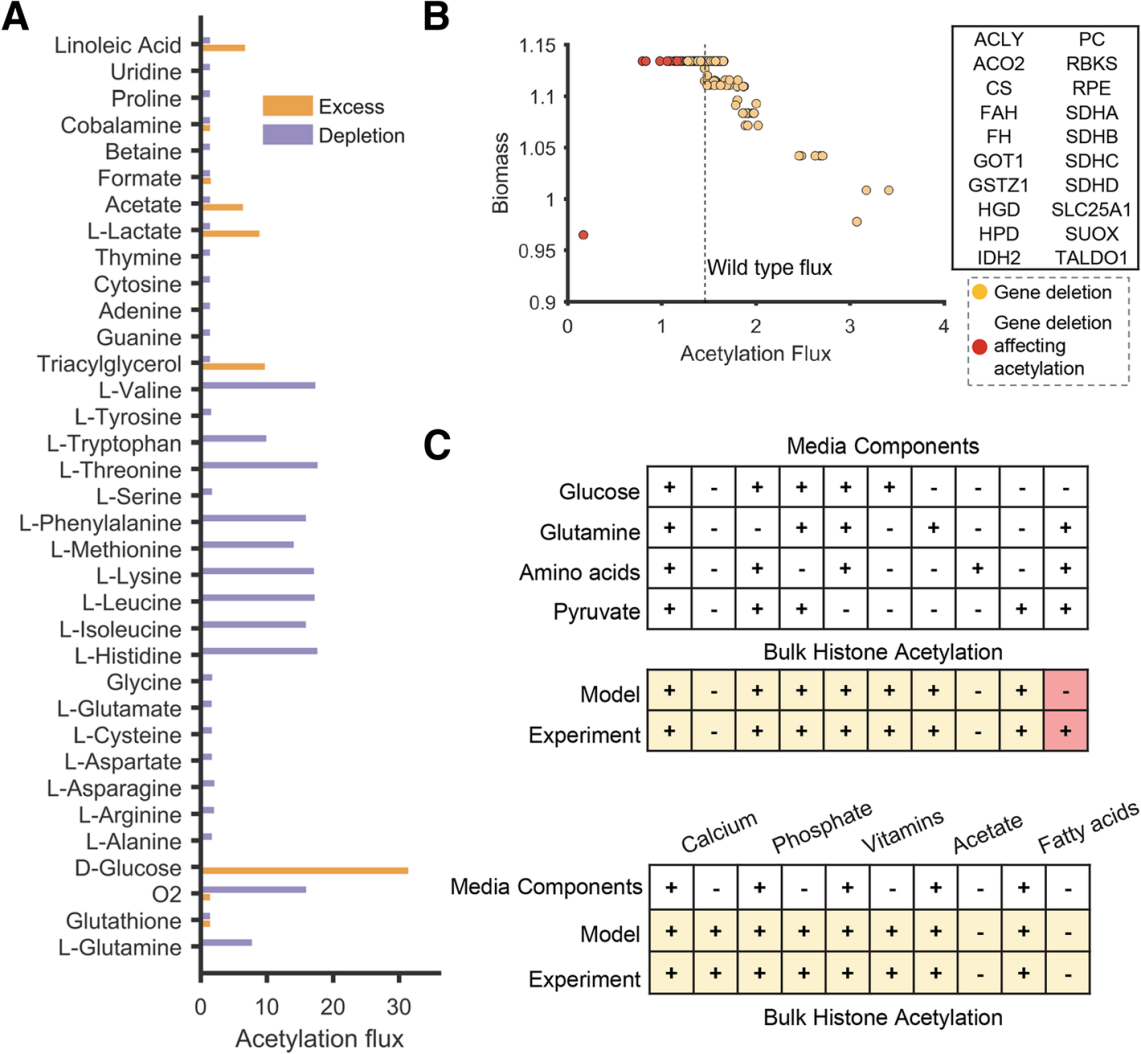
Predicting the effect of genetic deletion and nutrient changes on bulk acetylation using FBA. **a** The bar chart depicts the impact of reducing (purple bars) or increasing (orange bars) the level of metabolites in the culture media on acetylation flux (mmol/h per gram dry weight). **b** The plot shows the effect of gene deletions on bulk acetylation. The impact of gene deletion on the biomass production was used as a counter-screen to identify those genes that specifically impact protein acetylation. Gene deletions that exhibit at least 50% of the wild-type biomass production rate are shown. The top 20 genes that preferentially affect acetylation over growth are highlighted as red markers and displayed in the table. **c** Our model correctly predicted the impact of removing amino acids, pyruvate, glucose, acetate, fatty acids, vitamins, and minerals from the media on acetylation. The plus/minus sign indicates the presence or absence of specific nutrients in the media. Conditions predicted to have less than 5% of the wild-type acetylation flux by the model were assumed to be not supporting acetylation. Conditions experimentally observed to support (+) or not support (−) acetylation are also denoted by a plus or minus sign. The condition with incorrect prediction is shown in red. This incorrect prediction was resolved by accounting for trace nutrients in serum (Additional file 1: Figure S1)

To predict the impact of the cellular metabolic state on acetylation, we subjected the metabolic model to different nutrient environments and simulated the impact on acetylation (Figs. 1 and 2a). Specifically, the effect of addition or removal of glucose, glutamine, essential amino acids, and trace nutrients on acetylation was simulated using flux balance analysis (FBA) [20]. FBA identifies an optimal flux through the metabolic network that maximizes an objective. In this case, we use the commonly used FBA objective, the maximization of biomass production. We also included the flux through the protein acetylation reaction as a secondary FBA objective, i.e., it has a relatively small optimization weight compared to biomass. The underlying assumption is that acetylation will consume a small fraction of the cellular resources compared to biomass synthesis (Methods). To support this assumption, we used data from total potential acetylation sites in the human genome [13] and experimental data on carbon flux tracing [21]. We mathematically estimated that diverting a small fraction (< 5%) of the cytoplasmic flux of acetyl-CoA would be sufficient to acetylate all the sites in a cell in 24 h (Methods).

The metabolic model recapitulated known acetylation changes occurring due to a shift in the metabolic state. Switching to high or low glucose significantly affected acetylation levels, which are known to change with glucose flux [4, 19] (Fig. 2a). Bulk histone acetylation in yeast cells has also been shown to be responsive to glucose availability [3]. Addition of acetate, hypoxia, and starvation of amino acids also increased acetylation flux, consistent with experimental observations [19, 22–26]. To further validate the impact of media components on acetylation predicted by the model, we compared predictions with data from recent biochemical studies [19, 27–29] (Fig. 2c). Specifically, these studies measured the impact of addition or removal of amino acids, glucose, acetate, minerals, and vitamins on histone acetylation in cancer cell lines. Our model correctly predicted the impact of 19 out of the 20 nutrient changes. These results suggest that acetylation levels can be predicted based on the carbon flux and energetic state of the cell.

The ability of the model to reproduce these diverse observations provided new insights on the interaction between acetylation and metabolism. Our analysis suggests that bulk acetylation reflects excess carbon flux that is not used for biomass synthesis. Hence, the model predicted acetylation even in the absence of growth, which was observed experimentally in conditions with a carbon source but without a nitrogen source (i.e., depletion of amino acids). In contrast, growth conditions without excess carbon, such as those with amino acids alone, did not display histone acetylation (Fig. 2c).

The inconsistent observation between the model and experiment arose in a media condition lacking the carbon source—glucose. The model did not predict any acetylation, while it was observed experimentally. Acetylation in this condition was only possible in the model if there was an additional carbon source such as acetate or fatty acids. A recent study has found significant levels of acetate in DMEM media, which is usually not accounted for in biochemical studies [30]. This acetate can replenish cellular acetyl-CoA pools [30], and our modeling suggests that this can potentially support histone acetylation. We then re-evaluated the model predictions after including all nutrients that can be present in trace amounts in serum—amino acids from uptake or degradation of serum proteins, trace amounts of fatty acids, and acetate. These components are sufficient to support acetylation in this condition that contained all major nutrients except glucose. However, the trace nutrients were insufficient to promote acetylation if no major nutrients were present (Additional file 1: Figure S1). Thus, this inconsistent prediction points to the potential role of additional components in culture media that can impact acetylation.

### Metabolic modeling predicts bulk histone acetylation levels in diverse cancer cell lines

Since FBA correctly identified the impact of distinct metabolic conditions on acetylation levels, we next tested if FBA can predict more nuanced differences in the basal metabolic state and acetylation levels between different cancer cell lines. We used bulk histone acetylation levels from LeRoy et al.’s study, which measured acetylation levels using quantitative proteomics across a panel of diverse cell lines representing lung, liver, colon, and brain tumors [31].

To predict cell-type-specific changes in acetylation levels due to their underlying metabolism, we inferred their metabolic state from gene expression data [32]. Metabolic network models have been applied successfully to predict metabolic behaviors of human tissues and cancer cells using transcriptomic data [33–36]. We obtained gene expression data for the cell lines in LeRoy et al. study from the Cancer Cell Line Encyclopedia (CCLE) project [37] and integrated it with the human metabolic network model using the iMAT approach [32] (Methods).

**Fig. 3:**
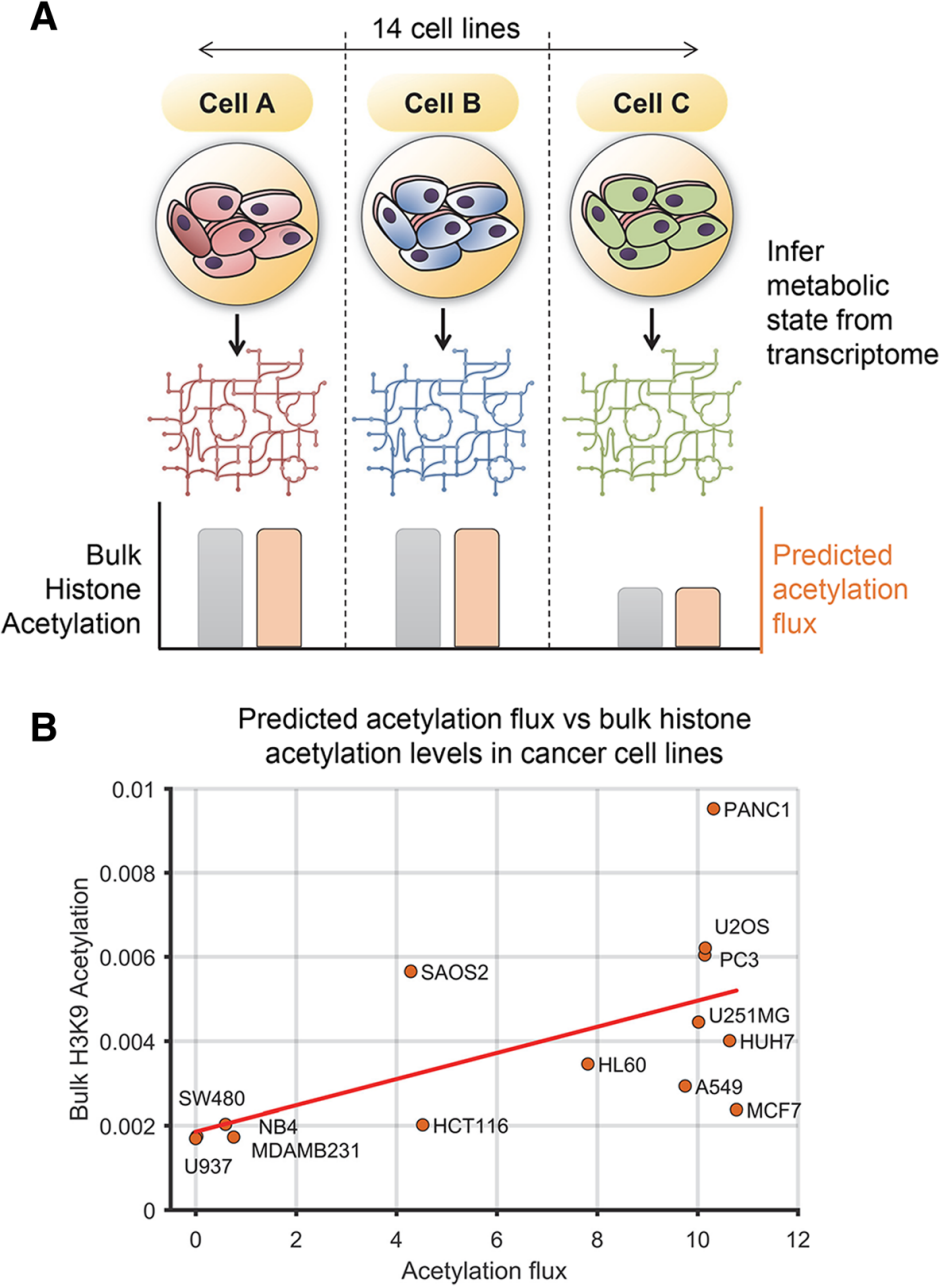
Predicting the effect of the basal metabolic state on histone acetylation levels using FBA. **a** Schematic of the approach to infer the basal metabolic state using transcriptomic data. The iMAT approach was used to integrate transcriptome data from a panel of cancer cell lines with the human metabolic network model to infer cell-line-specific metabolic state and acetylation flux. **b** The scatter plot shows the predicted acetylation flux (mmol/gDW cells/h) in each cell line and the bulk histone H3K9 acetylation levels from proteomics. The acetylation flux correlated significantly with the level of bulk histone H3K9 acetylation in these cell lines. The acetylation flux in the high flux group of cell lines is constrained by the availability of carbon units

The transcriptional state of each cell line resulted in distinct metabolic states and acetylation levels. The predicted acetylation flux of these 14 cell lines was compared to the net acetylation levels measured using proteomics in these cell lines. The acetylation levels predicted by the model correlated significantly across all the cell lines with bulk H3K9 acetylation levels, the major acetylation mark known to be sensitive to metabolism (Pearson’s correlation *R* = 0.6, *p* value = 0.022, FDR < 0.05, Fig. 3). However, there was low non-significant correlation with other acetylation marks (H3K27, *R* = 0.25, and H3K23, *R* = 0.35). Given the small sample size of our dataset (14 cell lines), the predictive power on other acetylation marks might be discernable in a larger dataset in the future. Overall, our analysis suggests that genome-scale modeling can predict the impact of the cellular metabolic state on bulk histone H3K9 acetylation.

### Predicting metabolic enzymes that affect protein acetylation

We next performed gene deletion analysis to discover metabolic genes that can impact acetylation. We systematically knocked out all 1487 metabolic genes in our model and quantified the effect on protein acetylation (Fig. 2b). This analysis identified 24 metabolic genes that reduced acetylation flux by 1% or more relative to wild type (hereafter referred to as acetylation-impacting genes). This includes those known to affect acetylation such as enzyme ATP citrate lyase (ACLY), TCA cycle genes such as IDH and SDH, and the mitochondrial citrate transporter SLC25A1 (Table 1). Cells lacking the ACLY displayed severely reduced levels of bulk histone acetylation [24]. In addition to the 24 acetylation-impacting genes, we also identified 39 genes that increased acetylation when deleted (hereafter referred to as acetylation-enhancing genes). This set of 39 genes is involved in reactions that consume acetyl-CoA. These include reactions in fatty acid synthesis, one-carbon metabolism, and amino acid synthesis that consume carbon units (Additional file 1: Table S1).

**Table 1:** Functional significance of the 24 metabolic genes predicted by the model to impact acetylation

Gene	Gene description	1. Acetylated?	2. Interacts with acetylase?	3. Interacts with deacetylase?	4. Sirtuin target?
AASS	Aminoadipate-semialdehyde synthase	X			
ACLY	ATP citrate lyase	X	X	X	
ACO2	Aconitase 2, mitochondrial	X	X	X	
ALDH1L1	Aldehyde dehydrogenase 1 family, member L1	X			
CS	Citrate synthase	X	X	X	
FAH	Fumarylacetoacetate hydrolase (fumarylacetoacetase)	X			
FH	Fumarate hydratase	X	X	X	
GOT1	Glutamic-oxaloacetic transaminase 1, soluble (aspartate aminotransferase 1)	X			
GSTZ1	Glutathione transferase zeta 1	X			
HGD	Homogentisate 1,2-dioxygenase	X			
HPD	4-Hydroxyphenylpyruvate dioxygenase				
IDH2	Isocitrate dehydrogenase 2 (NADP+), mitochondrial	X		X	X
PC	Pyruvate carboxylase	X	X		
OAT	Ornithine aminotransferase	X	X	X	
RBKS	Ribokinase				
RPE	Ribulose-5-phosphate-3-epimerase				
SDHA	Succinate dehydrogenase complex, subunit A, flavoprotein	X	X	X	X
SDHB	Succinate dehydrogenase complex, subunit B, iron sulfur (Ip)		X	X	X
SDHC	Succinate dehydrogenase complex, subunit C				X
SDHD	Succinate dehydrogenase complex, subunit D				X
SHMT1	Serine hydroxymethyltransferase 1 (soluble)	X	X		
SLC25A1	Solute carrier family 25 (mitochondrial carrier; citrate transporter)	X	X	X	
SUOX	Sulfite oxidase	X			
TALDO1	Transaldolase 1	X	X		

Column 1 shows acetylation status based on protein acetylation database. Column 2 shows proteins that are found to physically interact with an acetylase. Column 3 shows proteins that are found to physically interact with a deacetylase enzyme. Column 4 shows predicted targets of sirtuins, a major class of deacetylases (X mark implies true)

Our analysis uncovered several novel enzymes in addition to those that have been previously reported to impact acetylation. To assess the biological significance of this prediction, we compared the 24 acetylation-impacting metabolic genes with data from the protein acetylation database [38], which catalogs all known acetylation targets in humans. Proteomic studies have identified several metabolic enzymes as targets of acetylation [6, 7, 39]. Proteins encoded by 18 of the 24 acetylation-impacting genes were themselves targets of protein acetylation. In addition, a significant fraction of the enzymes predicted to affect acetylation by the model were found to be direct targets of Sirtuins, a major class of deacetylases. The fraction of enzymes that were found to be acetylated and deacetylated among those predicted by the metabolic model were significantly higher than would be expected by chance (*p* value = 8 × 10^− 15^ for acetylation and *p* value = 9 × 10^− 6^ for deacetylation respectively; Table 1). Intriguingly, none of the 39 acetylation-increasing genes were targets of Sirtuins, suggesting that Sirtuins regulate reactions involved in production but not the consumption of acetyl units.

To further corroborate our predictions of enzymes that impact acetylation, physical interaction data from literature were mined for associations between metabolic enzymes and acetylation machinery. Proteins involved in the same cellular process are more likely to physically interact [40]. We hypothesized that the metabolic enzymes identified by our model to affect acetylation levels will have strong physical interactions with acetylation or deacetylated-related enzymes.

Using data from the Biogrid database [41], which contains physical interaction data curated from literature, interactions between metabolic and acetylation-related enzymes in human cells were identified. Several metabolic enzymes predicted by our model to influence acetylation interacted physically with either a protein acetylase or deacetylase enzyme (Additional file 1: Table S2, Table S3). This overlap is significantly higher than expected by randomly choosing a metabolic enzyme (*p* value = 0.01 and 0.002 for acetylation-impacting and acetylation-enhancing enzymes respectively, hypergeometric test). Further, we compared the extent of overlap with acetylases and deacetylases separately. We observed a significant overlap between acetylation-enhancing genes with protein deacetylases but not with acetylases (*p* value = 0.0004 and 0.07 respectively). In contrast, acetylation-impacting genes have a significant number of interactions with both protein acetylases and deacetylases (*p* value = 0.003 and 0.005 respectively). Thus, acetylases predominantly interact with enzymes that lead to the synthesis of acetyl units. For example, the acetylation-impacting enzyme ACLY had physical interactions with several acetylases. ACLY is known to be transported to the nucleus to facilitate acetylation [42, 43]. The physical interaction suggests that the product of the ACLY metabolic reaction (acetyl-CoA) can be directly channeled as substrate for acetylases. We also found physical interactions between the TCA cycle enzymes citrate synthase (CS) and succinate dehydrogenase (SDH) with deacetylase enzymes HDAC5 and SIRT7 respectively. Overall, our results suggest that enzymes identified by our model to impact acetylation show strong functional association with protein acetylation pathways, thus corroborating our predictions.

### Altering sensitivity of cancer cells to protein deacetylase inhibitors by changing their metabolic state

Our analysis so far suggests that genome-scale modeling can predict the impact of the metabolic state on global protein acetylation. We next predicted the impact of the cellular metabolic state on susceptibility to drugs that disrupt cellular protein acetylation, specifically protein deacetylase inhibitors. The human genome encodes 18 different lysine deacetylases (KDACs) that regulate numerous biochemical pathways [7]. KDACs are considered attractive therapeutic targets for treating not just cancers, but also viral infections, inflammation, neurodegenerative diseases, and metabolic disorders [10]. Four deacetylase inhibitors (vorinostat, romidepsin, belinostat, panobinostat) are approved clinically for cancer treatment [44]. However, identifying tumors that are sensitive to these drugs is a significant clinical challenge [45, 46].

Deacetylase enzymes catalyze the removal of acetylation marks from histones and other proteins. They are sensitive to, and directly impact, the metabolic state of the cell [47]. Deacetylase inhibitors block the deacetylation of proteins and are predicted to cause cell death by increasing histone acetylation and triggering apoptosis [45, 46, 48]. Hence, we hypothesized that conditions that favor increased acetylation should enhance sensitivity to these drugs.

Since genome-scale modeling allowed us to predict metabolic conditions that increased protein acetylation, we next used it to predict conditions that enhance sensitivity to deacetylase inhibitors. Specifically, we explored the use of metabolic network modeling to identify cell types that are sensitive to KDAC inhibition based on their metabolic and acetylation state. This can enable in silico identification of tumors most sensitive to these drugs.

To validate our hypothesis, we experimentally measured the sensitivity of the ovarian cancer cell line (HeLa) to vorinostat (also known as suberoylanilide hydroxamic acid (SAHA)). Vorinostat was the first approved histone deacetylase inhibitor. HeLa cells were cultured in 92 different metabolic conditions using Biolog phenotype microarrays and exposed to vorinostat or control (DMSO). The culture conditions in the Biolog arrays included a wide range of carbon sources that result in distinct patterns of metabolic activity in the cell [49]. This broad range of metabolic activity allowed us to assess the impact of the metabolic state of the cell on vorinostat sensitivity.

**Fig. 4:**
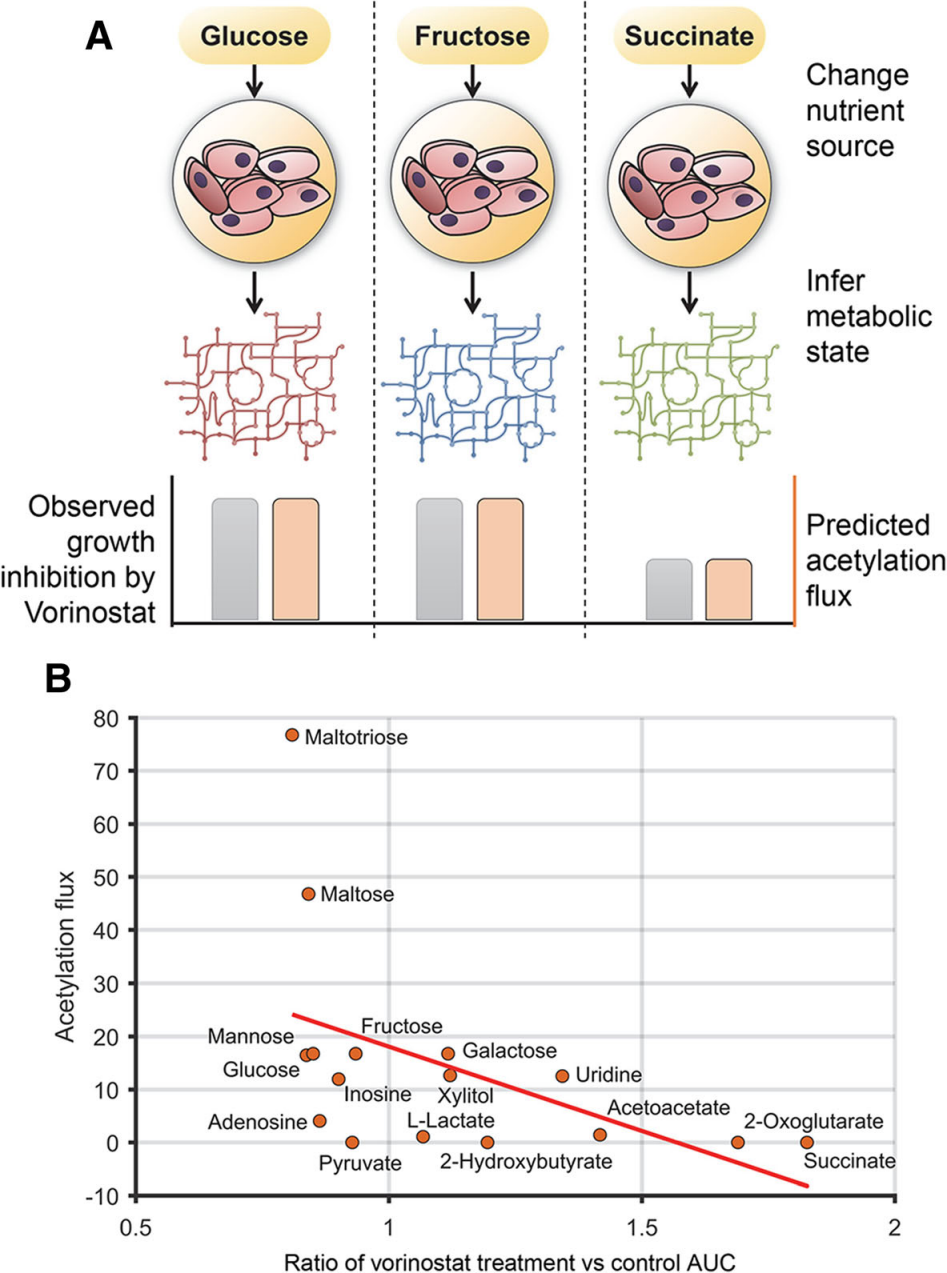
Changing the metabolic state predictably alters sensitivity to the deacetylase inhibitor—vorinostat. **a** Schematic of the phenotype microarray analysis. HeLa cells were cultured in different metabolic conditions and exposed to vorinostat or control (DMSO). The metabolic state and acetylation flux in each condition were determined using FBA. **b** The acetylation flux (mmol/gDW cells/h) correlated significantly with the extent of growth inhibition by vorinostat. The scatter plot shows the predicted acetylation flux in each metabolic condition and the ratio of the area under the growth curve (AUC) for vorinostat treatment relative to control. Conditions with lower ratio (< 1) were more sensitive to vorinostat and showed higher acetylation flux. Average of four replicates shown

The growth inhibition by vorinostat, quantified by area under the growth curve (AUC) relative to control, changed significantly with different nutrient conditions (Additional file 1: Table S4). Some growth conditions such as succinate as a primary carbon source were protective of vorinostat treatment, while growth in fructose or mannose enhanced sensitivity (Fig. 4). We then predicted the growth rate and acetylation flux in these conditions using metabolic modeling and compared the model predictions with sensitivity to vorinostat. Among all the conditions tested, we compared those substrates that supported growth in Biolog arrays and were part of the metabolic model. The acetylation flux predicted in each condition strongly correlated with vorinostat sensitivity (*R* = − 0.66, *p* value = 0.0051, Fig. 4; Additional file 1: Figure S2). Conditions that supported higher acetylation flux had greater growth inhibition, consistent with our hypothesis. In contrast, the predicted growth rate correlated weakly with sensitivity (*R* = − 0.3, *p* value > 0.05).

While the predicted sensitivity showed high concordance with experiments across most substrates, the model incorrectly over-predicted acetylation flux and corresponding sensitivity in conditions with maltose and galactose as carbon sources; galactose is metabolized differently despite similarity to glucose [50]. Intriguingly, vorinostat enhanced the growth of cells under some conditions. KDAC inhibitors are known to reduce glucose metabolism and increase mitochondrial oxidative metabolism [51]. This metabolic impact may be beneficial in conditions that lack glucose or other fermentable sugars. We observed a strong correlation between the extent of growth enhancement and acetylation flux predicted in those conditions (Additional file 1: Figure S2). Hence, KDAC inhibitors may enhance growth in conditions with low acetylation while inhibiting growth in conditions with high acetylation flux. This dependence on the cellular metabolic state may explain the growth-promoting effect of KDAC inhibitor treatment on stem cells and neurons [52, 53].

### Predicting cellular sensitivity to protein deacetylase inhibitors using the metabolic state

Since distinct metabolic conditions showed differences in sensitivity to vorinostat, we next tested if our model can predict more nuanced differences in metabolic activity between different cancer cell lines and their corresponding impact on sensitivity to KDAC inhibitors. Large-scale therapeutic screening data from Seashore-Ludlow et al. was used to identify variation in sensitivity to KDAC inhibitors among a panel of 860 cancer cell lines encompassing various tissue and tumor types [54]. The transcriptomic state of these cell lines has been characterized using microarrays as part of the Cancer Cell Line Encyclopedia (CCLE) project [37].

**Fig. 5:**
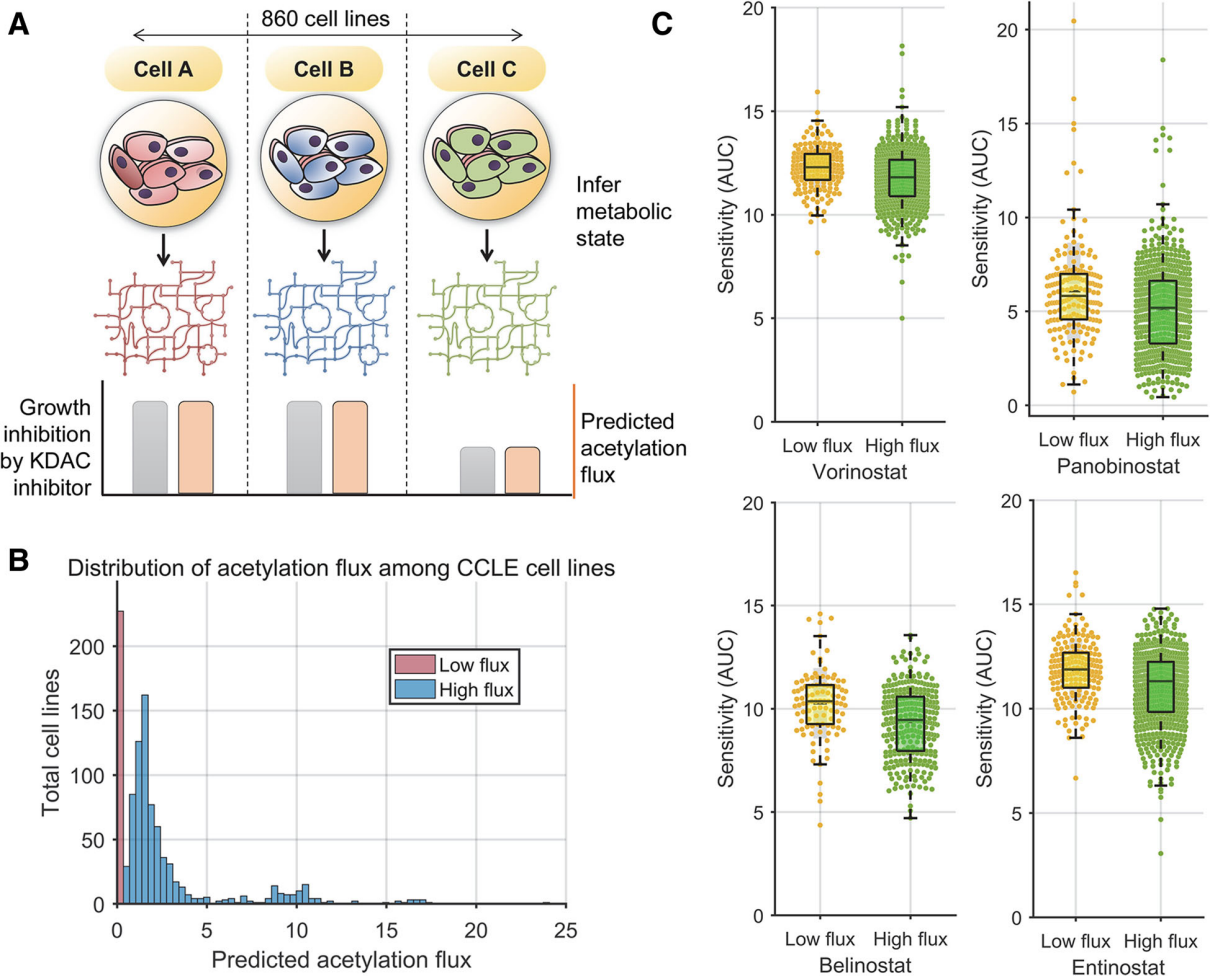
Predicting sensitivity to deacetylase inhibitors by inferring basal metabolic network state. **a** Schematic overview of the analysis comparing the sensitivity of cell lines from CCLE to deacetylase inhibitors and their corresponding metabolic state. **b** The histogram shows the distribution of predicted acetylation flux (mmol/gDW cells/h) among the CCLE cell lines. The data revealed two groups of cell lines—cell lines predicted to have no or very low acetylation flux (flux < 0.05) and a high flux group (flux > 0.05). **c** KDAC inhibitors, vorinostat, panobinostat, belinostat, and entinostat, were significantly more sensitive against the high flux group than the low flux group of cell lines (*p* value < 10^− 5^, *t*-test; Additional file 1: Figure S3). The bar graphs depict the area under the growth curve (AUC) of CCLE cell lines after treatment with the drugs, with lower AUC implying greater growth inhibition. The results are robust to the cut-offs for high and low acetylation flux group (Additional file 1: Figure S3)

We integrated gene expression data for the CCLE cell lines along with their growth media composition with the genome-scale human metabolic network model to infer their metabolic state using the iMAT approach (Methods). The sensitivity of these cell lines to vorinostat was compared to the net acetylation flux predicted by the model in each of these cell lines. Overall, the net acetylation flux in these cell lines was bimodal in nature with a very low and high flux group (Fig. 5b; Additional file 1: Figure S3). The high flux group showed higher expression levels of reactions associated with acetyl-CoA synthesis and fatty acid metabolism such as acetyl-CoA synthetase and propionyl-CoA carboxylase (Additional file 1: Table S5).

We compared the sensitivity of the cell lines with high acetylation flux state with those with low acetylation flux. Cell lines with high acetylation flux were found to be significantly more sensitive to vorinostat treatment than those with low acetylation flux (Fig. 5c; *p* value = 4 × 10^− 6^, *t*-test). This is consistent with the observation across metabolic conditions using Biolog phenotype arrays. The low throughput Biolog assays revealed a strong quantitative relationship with the model predictions. However, these were observed with a single cell line. In contrast, the high-throughput drug-screening assays from Seashore-Ludlow et al. involve hundreds of cell lines. Our results across this large dataset suggest that this is a statistically robust phenomenon.

To assess the generality of this observation to other KDAC inhibitors, we compared the pattern of sensitivity between high and low acetylation flux cell lines using data from three other clinically used KDAC inhibitors that were part of the Seashore-Ludlow et al. dataset—panobinostat, entinostat, and belinostat. Entinostat is currently in phase III clinical trials, and the other two drugs are FDA approved. All these drugs were significantly more sensitive to high acetylation flux state cell lines (*p* value < 10^− 6^, *t*-test; Fig. 5c, Additional file 1: Figure S3). Overall, these results suggest that the metabolic and acetylation state is predictive of sensitivity to KDAC inhibitors.

Finally, to assess the specificity of the acetylation flux metric in predicting sensitivity to KDAC inhibitors, we compared the differential sensitivity of all 481 compounds that were part of the Seashore-Ludlow et al. study between the high and low acetylation flux cell lines. KDAC inhibitors were significantly more strongly selective between the two groups than other drug classes (*p* value = 0.003, *t*-test; Additional file 1: Figure S4). This trend applies to both experimental and clinically used KDAC inhibitors (Additional file 1: Figure S4). Thus, the observed trend is not due to an indirect correlation with other cellular processes such as the growth rate. Indeed, we observed a stronger trend using the Seashore-Ludlow et al. data after correcting for doubling time of each cell type (*p* value = 7 × 10^− 9^, Additional file 1: Figure S5).

### Predicting variability between protein deacetylase inhibitors

So far, we have predicted the impact of the cellular metabolic state on sensitivity to protein deacetylase inhibitors. We next incorporated the direct impact of these drugs on cellular metabolism. Vorinostat treatment has been previously shown to reduce glucose and fatty acid metabolism in myeloma cell lines [51]. Incorporating the impact of these drugs on metabolism would allow us to predict differences in sensitivity resulting from both basal metabolic state and the unique impact of each KDAC inhibitor. KDAC inhibitors display variability in their sensitivity to different cell types [53]. KDAC inhibitors block distinct histone deacetylases resulting in transcriptomic changes that influence the levels of metabolic enzymes [48, 53].

To model the unique effect of each drug, we interpreted the pattern of differential gene expression from cells exposed to these drugs with metabolic network models. We used data measured in one representative cell line and used it to predict the metabolic impact of each KDAC inhibitor on all CCLE cell lines. We analyzed transcriptomic signatures of four different KDAC inhibitors (vorinostat, panobinostat, entinostat, and belinostat) before and after treatment from UKN1 cells [55]. Several metabolic genes were uniquely differentially expressed in response to each KDAC inhibitor, which may lead to variation in sensitivity (Additional file 1: Table S6). This list of downregulated and upregulated genes was overlaid onto all the CCLE cell line metabolic models to predict variation in drug sensitivities among the CCLE cell lines. The net acetylation flux due to both basal transcriptomic state and drug-induced transcriptomic changes were determined for all cell lines for the four KDAC inhibitors using the iMAT approach.

**Fig. 6:**
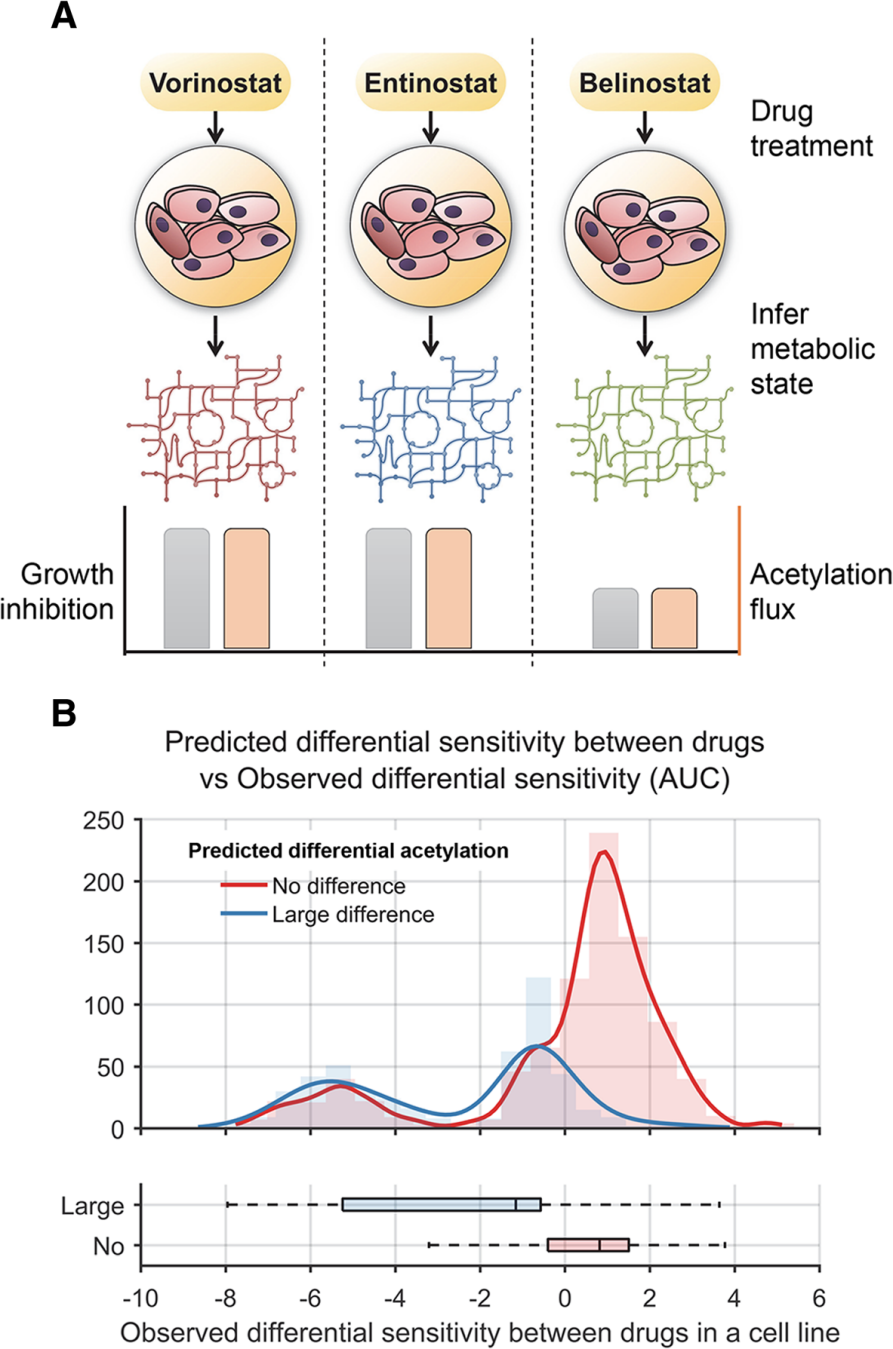
Metabolic rewiring by KDAC inhibitors predicts variation in sensitivity between these inhibitors among cancer cell lines. **a** Overview of the analysis comparing the sensitivity of CCLE cell lines to four KDAC inhibitors—vorinostat, panobinostat, belinostat, and entinostat. The acetylation flux in each cell line corresponding to each drug treatment was determined by FBA by accounting for both the basal transcriptomic profiles of each cell line and the metabolic impact of these drugs. Cell line-drug combinations were grouped into two classes—the first group of cell line-drug pairs did not show any significant difference in acetylation flux compared to the other drugs, while the second class showed large differences. **b** The histogram and box plots show the distribution of normalized sensitivity scores of the four drugs among the two cell line groups. The experimental sensitivity data was median normalized so that a score of 0 implies that there was no difference in sensitivity between the drugs while a negative score implies greater sensitivity for a drug relative to other three drugs. The normalized sensitivity scores were significantly lower for the group that showed large difference in acetylation flux between the drugs compared to the group that showed no acetylation flux difference (*p* value = 2 × 10^− 62^, *t*-test)

We compared the extent of acetylation flux change caused by each drug in a cell line to the sensitivity of each of these drugs. Cell lines showing higher acetylation flux reduction for a drug relative to others displayed significantly greater growth inhibition to that drug, with an average of 25.3% difference in growth (equivalent to 2.6 AUC units, *p* value = 10^− 62^, *t*-test, Fig. 6). Overall, cell lines with high basal acetylation flux are more sensitive to all KDAC inhibitors; further, the extent of acetylation flux reduction after drug treatment correlates with sensitivity to the corresponding KDAC inhibitor. Thus, combining the impact of basal metabolic state and the impact of KDAC-induced differential enzyme expression can predict both sensitivity and variability between KDAC inhibitors. In sum, our study provides a framework for predicting the potency of KDAC inhibitors from baseline (pre-treatment) transcriptomic profiles. Additional file 1: Figure S6 summarizes the steps for applying our approach for a new cell line or tumor sample.

## Discussion

In this study, we built a genome-scale biochemical model to predict the impact of cellular metabolic state and availability of nutrients on acetylation. A key observation from our study is that histone acetylation dynamics reflects the flux of excess carbon that is not used for biomass synthesis (overflow metabolism). Overflow metabolism has also been proposed to drive acetylation in prokaryotes [56, 57]. Conditions that lack amino acids or oxygen can increase acetylation as there is excess carbon available for acetylation from glucose. Strikingly, we observed acetylation even in certain nutrient starvation conditions that do not support growth, which is also observed experimentally (Fig. 2c).

Our analysis also identified known and novel metabolic enzymes that can impact acetylation. These predictions were supported by numerous physical interactions between the acetylation-impacting metabolic enzymes predicted by our model with proteins in the acetylation machinery. This suggests that the activity of these metabolic enzymes can impact protein acetylation and are in turn regulated by protein acetylation. This reciprocal regulation to maintain homeostasis has also been observed between metabolic pathways and signaling pathways [58–60] .

Since acetylation plays a central role in signaling and chromatin regulation, mutations in the acetylation-impacting metabolic genes can significantly affect cellular homeostasis. The acetylation-impacting genes, IDH2, SDHA/B/C, and FH, are annotated as being frequently mutated in cancers in the COSMIC database [61]. Aberrant expression of these genes may also lead to altered acetylation in tumors. The genes ACLY, CS, RPE, and IDH2 were found to be among the top 10% of the genes that were frequently overexpressed across 19 cancer types from a meta-analysis study of 1981 tumors (FDR *p* value < 0.05) [62]. These genes predicted by our model are potential drug targets for disorders with dysregulated epigenome.

We then demonstrated that our model can predict the impact of the metabolic state on sensitivity to deacetylase inhibitors. We hypothesized that conditions that favor increased acetylation should enhance sensitivity to these drugs, which cause cell death by blocking deacetylation. Consistent with this hypothesis, we were able to predict and validate both metabolic conditions and cell types that show increased sensitivity to these drugs. Our experimental screen across diverse metabolic conditions revealed that sensitivity to vorinostat changed significantly with the metabolic state. Notably, our biochemical model accurately predicted the sensitivity to vorinostat by predicting the acetylation flux in diverse nutrient conditions. Nutrients that were modeled in this study such as glucose, acetate, amino acids, and lactate are also metabolized by tumors in vivo [63, 64]. While glucose is the common carbon source, some tumors also use lactate [64]. Our analysis suggests that while glucose condition increases sensitivity, growth in lactate may offer protection against vorinostat. Hence, this drug may be effective only against some tumor types. This is potentially relevant for targeting cancers with distinct metabolic states [65]. While the tumor microenvironment is complex and dynamic, our ability to predict the impact of drug efficacy in simple defined environments is the first step towards addressing the in vivo complexity. Our study paves the way for dynamic models that can simulate the impact of changing the metabolic conditions.

Analysis of KDAC inhibitor sensitivity across 860 cancer cell lines revealed that differences in basal metabolic state among cells can lead to variation in sensitivity. Using metabolic modeling, we were able to group cell lines based on their acetylation state and predict those that show increased sensitivity to deacetylase inhibition. Identifying tumors that are sensitive to specific KDAC inhibitors is a significant clinical challenge [46]. Our approach can help predict efficacy of deacetylase inhibitors against different cancer types using transcriptomic data. Transcriptomic profiling is increasingly done for tumors for treatment stratification and prognosis [66]. A very small tissue sample is needed for transcriptomics compared to the requirements for cell culture and drug screening.

Furthermore, unique differences in the transcriptomic pattern induced by each KDAC inhibitor correlate with variation in sensitivity between these inhibitors. By analyzing sensitivity to four clinically used KDAC inhibitors across all CCLE cell lines, we found that cell lines that showed higher acetylation flux reduction after treatment with a KDAC inhibitor relative to others were more sensitive to the corresponding drug. Our approach complements machine-learning and high-throughput screening approaches [67] by providing a rational mechanistic strategy to predict and tune sensitivity of KDAC inhibitors based on acetylation flux. Genome-scale modeling has been shown to accurately predict the metabolic state of cancer cells from transcriptomic data. Future analysis using proteomics and metabolomics data could lead to tumor stratification with greater accuracy.

While we have focused on the impact of metabolism and nutrition on acetylation in this study, the activity of acetylases and metabolic enzymes can also be regulated by signaling pathways which can subsequently impact bulk acetylation levels [7]. Further, while our model predicts bulk acetylation levels, it does not provide insights on acetylation changes in specific protein sites. In future, knowledge of specific targets, binding affinity, and expression levels of acetylation and deacetylation enzymes can enable prediction of acetylation dynamics at specific sites in the proteome. Similarly, incorporating the role of redox factors, FADH and NADH, that can influence activity of deacetylases can potentially resolve inconsistencies observed in nutrient conditions such as galactose media that impact redox state [68]. Similar to acetylation, which is sensitive to the flux of acetyl-CoA, recent studies have revealed that histone methylation is sensitive to the flux of the substrate S-Adenosyl Methionine (SAM) [17, 69]. Our study can pave the way for modeling other histone modifications like acylation, succinylation, and methylation that also depend on metabolic intermediates.

## Conclusion

Histone acetylation links gene regulation with metabolism, and aberrant acetylation is associated with cancers and metabolic disorders. Here we developed a genome-scale biochemical network model to predict the impact of metabolism on acetylation. Our model revealed that histone acetylation levels change predictably depending on cellular carbon and nutrient availability. We identified several novel metabolic genes that can impact acetylation when deleted. Our approach provided two key insights related to cellular metabolism and sensitivity to deacetylase inhibition. Firstly, our experimental screen across diverse metabolic conditions and analysis across 860 cancer cell lines revealed that sensitivity to deacetylase inhibitors can be predicted from the cellular metabolic state. Secondly, unique rewiring of metabolic pathways induced by each KDAC inhibitor correlates with variability between these inhibitors. This is potentially relevant for tumor treatment stratification based on the metabolic state. Overall, our study demonstrates the strong and predictable interconnection between metabolism and acetylation.

## Methods

### Flux balance analysis (FBA)

In FBA, an optimal metabolic flux state is determined that maximizes an objective (usually the biomass). We used the biomass composition from Shlomi et al. [70] for the growth objective function. The biomass objective function represents the steady-state consumption of 42 essential metabolites, such as nucleotides, amino acids, and lipids required for cellular proliferation. In addition to maximizing the objective, the following constraints are satisfied—(1) the system is at steady state with mass balanced for all species (i.e., no accumulation or depletion of any intracellular metabolites in the network) and (2) the specified upper and lower bounds for each reaction in the network are not violated. FBA was used for simulating the impact of gene deletions and metabolic environment on protein acetylation. A total of 50 nutrients commonly present in rich media culture were used as starting points for nutrient addition in excess and nutrient removal.

FBA was performed in different nutrient environments with the protein acetylation reaction as the secondary objective (Optimization objective: maximize Biomass_reaction + epsilon × acetylation_reaction). Acetylation flux is optimized after biomass synthesis is maximized. We used a parameter (“epsilon”) to incorporate the relative weights for optimizing biomass flux relative to acetylation. Changing the value of epsilon (i.e., relative weights) over a 10,000-fold range from 0.01 to 10^− 6^ does not influence the value of acetylation flux and the accuracy of our approach (Additional file 1: Figure S7, Additional file 1: Table S7). The two objectives optimized here can be conflicting or potentially independent depending on nutrient availability. Since acetylation flux is a secondary objective for our optimization, any value of epsilon that is relatively small compared to biomass function would lead to the same value of acetylation flux. The mathematical approach used here with separate weights for different objectives is the most common way of solving such multi-objective problems [71]. This approach is scalable to any number of objectives.

### Total carbon flux necessary for acetylation compared to other biosynthetic processes

Using data from total potential acetylation sites in the human genome [13], we find that diverting a small fraction of the cytoplasmic flux of acetyl-CoA would be sufficient to acetylate all the sites in a cell. The observed flux through cytosolic acetyl-CoA synthesis reaction in human iBMK cells is 2 nmol/h/μlcells [21], which is equivalent to 10^9^ molecules per hour in each cell. Assuming a total of 10^9^ acetylation sites in a mammalian cell [13], redirecting 1/24th of the cytosolic acetyl-CoA production flux towards the nucleus in a period of 24 h can be sufficient to saturate all the histone acetylation sites, assuming a relatively low rate of deacetylation [63]. The mitochondrial carbon flux towards acetyl-CoA is 10-fold higher than cytosolic flux [21]. This suggests that redirection of carbon flux towards acetylation will not significantly impact biomass synthesis.

### Transcriptomic data integration

To integrate transcriptomic data with the metabolic network model, we used a linear version of the iMAT approach [32] implemented in the PROM algorithm [72]. In the iMAT approach, the number of active reactions associated with genes that are upregulated or highly expressed is maximized, while flux through reactions associated with downregulated reactions are minimized. The linear optimization version of the iMAT approach allows for continuous restriction of fluxes instead of setting reactions to be completely on or off [72]. The constraints are flexible, i.e., they allow for violation of individual constraints in order to maximize the overall consistency with the data. These inconsistencies may represent sites of regulation by other regulatory mechanisms [32]. We used this approach to infer a cell-type-specific metabolic state using transcriptomic data for CCLE cell lines and to incorporate transcriptomic evidence from KDAC inhibitor profiling data. The optimization problem was solved using the Gurobi mathematical programming solver. The entire series of steps for predicting acetylation flux from transcriptomic data is outlined in Additional file 1: Figure S8 and Additional file 1: Figure S9.

To assess the robustness of our results to the method of transcriptome integration, we repeated all our analyses with an alternative approach for transcriptomic data integration called GIMME [73]. This approach differs from the iMAT approach in that it assumes a direct linear association between extent of downregulation of a gene’s expression and the downregulation of flux through the corresponding enzyme encoded by it. We found that our results from this approach are consistent with our results using iMAT (Additional file 1: Table S8).

### Small molecule therapeutic screening data

Area under the growth curve (AUC) values for the small molecule therapeutic screening data for 481 compounds against 860 cell lines were obtained from Seashore-Ludlow et al. along with media used for each of the 860 cell lines. The media composition and transcriptomic data for these cell lines from CCLE was used to infer the metabolic network state for these cell lines as described above. In order to estimate the impact of the cellular doubling rate on drug sensitivity, we also used data from Hafner et al. that controls for this effect [74]. The growth-controlled efficacy metric was found to be superior to existing metrics for assessing the efficacy of drugs in dividing cells. Cell lines with missing growth inhibition values for the KDAC inhibitors were removed from the statistical analysis.

### Biolog phenotype microarray (PM) measurement

The phenotype microarray (PM) plates from Biolog Inc. (Hayward, CA, USA) contain diverse energy sources to profile the metabolic capabilities of a cell. Each well in a PM plate contains a single chemical as the sole energy source and production of NADH per well is monitored using a colorimetric redox dye chemistry. As the cells metabolize the energy source, tetrazolium dye in the media is reduced, producing a purple color according to the amount of NADH generated. The PM-M1 plate used in this study contains 92 different carbon energy sources along with negative control wells.

Biolog PM assays were performed as described in Boccuto et al. [75]. Viable cells were counted utilizing a TC20™ Automated Cell Counter. PM-M1 plates were incubated with 20,000 viable cells per well in a volume of 50 μL. The cells were incubated at 37 °C in 5% CO_2_, using the modified Biolog IF-M1 medium [75]. HeLa cells were exposed to vorinostat (2 μM) or DMSO (control) at the time they were added to the Biolog PM plates, for a 72-h exposure. The experiment was conducted in four replicates using the PM-M1 plate. During the final 24 h of exposure, the optical density of each well was measured every 15 min. The optical readings were normalized using quadruplicate readings from an empty plate (plates run with no cells, just media and dye). The normalized optical density readings were used for statistical analysis.

To control for the impact of slow or no growth, we used only those conditions that supported any growth above negative control (Background). Using various thresholds for growth did not impact the strong correlation between the predicted acetylation flux and growth inhibition (Additional file 1: Figure S2).

### Statistical analysis

The significance of overlap between gene lists was estimated using the hypergeometric test in MATLAB. Biolog data analysis was performed in R.

## Additional file

Additional file 1: File contains Supplementary figures 1-9 and Supplementary tables 1-8. (PDF 1.8 MB)
