# Supplementary material for: Genome-scale network model of metabolism and histone acetylation reveals metabolic dependencies of histone deacetylase inhibitors
Source: Genome Biol. 2019 Mar 1;20:49. doi: 10.1186/s13059-019-1661-z (PMC6397465; doi:10.1186/s13059-019-1661-z)
Supplement: Supplementary file 1 — File contains Supplementary figures 1-9 and Supplementary tables 1-8. (PDF 1.8 MB) [file 13059_2019_1661_MOESM1_ESM.pdf]

Supplementary Information for

Genome-scale network model of metabolism and histone acetylation reveals metabolic dependencies of histone deacetylase inhibitors

Media Components

Glucose	+	-	+	+	+	+	-	-	-	-
Glutamine	+	-	-	+	+	-	+	-	-	+
Amino acids	+	-	+	-	+	-	-	+	-	+
Pyruvate	+	-	+	+	-	-	-	-	+	+
**Trace serum nutrients**	+	+	+	+	+	+	+	+	+	+

Bulk Histone Acetylation

Model	14.0	0.0	20.4	28.7	1.5	15.0	1.1	0.0	11.7	0.0
Model with serum	14.0	0.0	20.4	28.7	1.5	7.3	11.1	0.0	12.1	4.2
Experiment	+	-	+	+	+	+	+	-	+	+

Media Components

	Calcium		Phosphate		Vitamins		Acetate		Fatty acids	
	+	-	+	-	+	-	+	-	+	-
**Trace serum nutrients**	+	+	+	+	+	+	+	+	+	+
Model	1.5	1.5	1.5	1.5	1.5	1.5	0.9	0.0	28.6	0.0
Model with serum	1.5	1.5	1.5	1.5	1.5	1.5	0.9	0.0	28.6	0.0
Experiment	+	+	+	+	+	+	+	-	+	-

Bulk Histone Acetylation

Fig. S1. Impact of nutrient sources and trace serum nutrients on acetylation. We re-evaluated the model predictions on the impact of nutrient sources in Figure 2C to investigate the effect of trace nutrients in serum (namely acetate, fatty acids and amino acids from albumin). The plus/minus sign indicates the presence or absence of specific nutrients in the media. Our model correctly predicted the impact of removing amino acids, glutamine, pyruvate and glucose from the media on acetylation (top panel). The bottom panel shows the impact of removing minerals (Calcium, Phosphate), vitamins, presence and absence of acetate and fatty acids as sole carbon sources on bulk acetylation. The condition with the incorrect prediction (shown in green) was resolved with the addition of nutrients from serum such as acetate or fatty acids. The plus/minus sign in the Experiment rows indicates conditions experimentally observed to support (+) or not support (-) acetylation. The acetylation flux predicted by the model in these conditions are also listed. Conditions predicted to have less than 5% of the wild type acetylation flux (1.5 mmol/gDW cells/hr) were assumed to be not supporting acetylation and are highlighted in red.

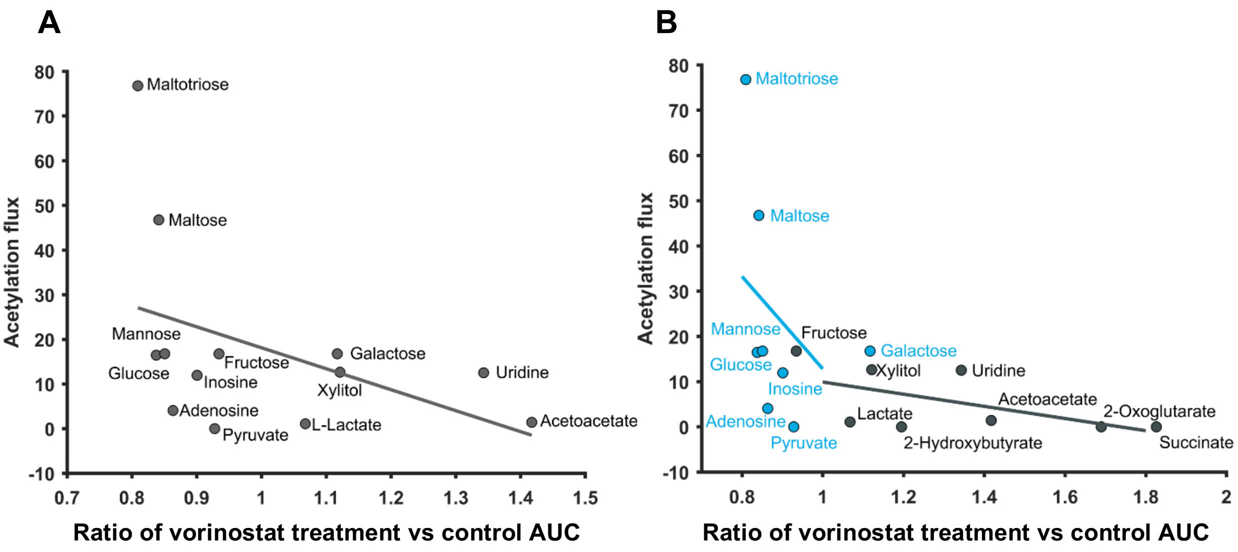

Fig. S2: The acetylation flux (mmol/gDW cells/hr) in each metabolic condition is predictive of sensitivity to vorinostat. **A.** Robustness of the correlation between acetylation flux and sensitivity to vorinostat to the choice of threshold for identifying conditions with viable growth in the Biolog assay. We used all conditions that showed significantly higher growth than negative control (AUC > 2) for estimating the correlation in Figure 4B. Panel A shows the correlation involving conditions with a higher growth threshold (AUC > 3) (R = -0.52). Our analysis at other thresholds also showed significant correlation between model prediction and experimental observations (R < -0.45). **B.** Dividing the conditions as those supporting high growth (AUC > 8, shown in blue) and low growth (2 < AUC < 8) also showed significant correlation between model prediction and experimental observations (R = -0.6 and -0.65 respectively).

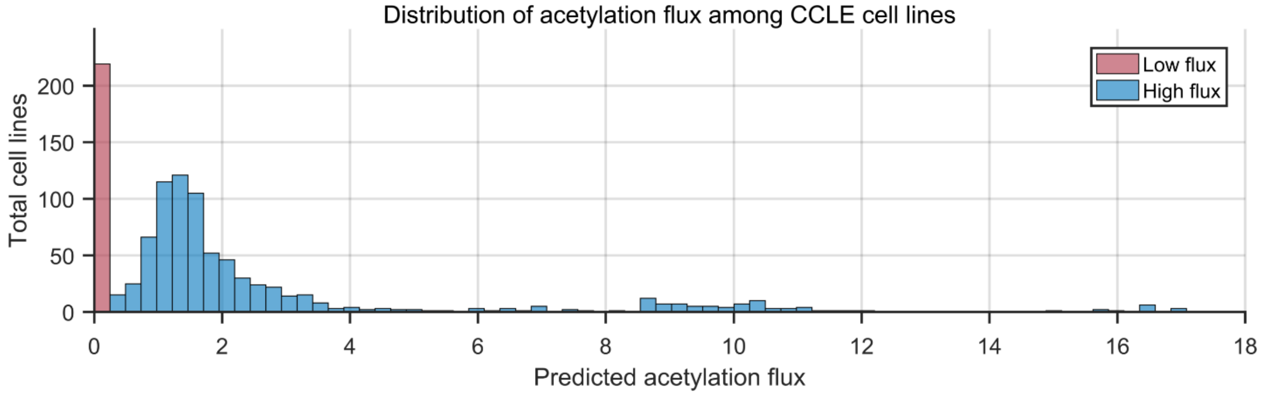

Drug\Threshold	0.05	0.64	1.3
Panobinostat	4.06×10^{-6}	7.37×10^{-6}	3.13×10^{-4}
Vorinostat	4.18×10^{-6}	2.85×10^{-5}	9.02×10^{-6}
Belinostat	3.05×10^{-9}	4.85×10^{-8}	3.74×10^{-4}
Entinostat	4.87×10^{-6}	5.83×10^{-6}	3.26×10^{-5}

Fig. S3: Impact of changing the acetylation flux cut-offs for classifying high and low acetylation flux cell lines. The histogram shows the distribution of predicted acetylation flux (mmol/gDW cells/hr) among the CCLE cell lines. The data revealed two groups of cell lines - cell lines predicted to have no or very low acetylation flux (flux < 0.05) and a high flux group (flux > 0.05). KDAC inhibitors – Vorinostat, Panobinostat, Belinostat and Entinostat, were significantly more sensitive against the high flux group than the low flux group of cell lines. We assessed the difference in sensitivity scores (AUC values) across the two groups using a t-test. The table shows the t-test p-values for each drug using various flux cut-offs for grouping the cell lines (0.05, 0.64 (25th percentile) and 1.3 (50th percentile). The results are robust to the cut-offs used for determining high and low acetylation flux group.

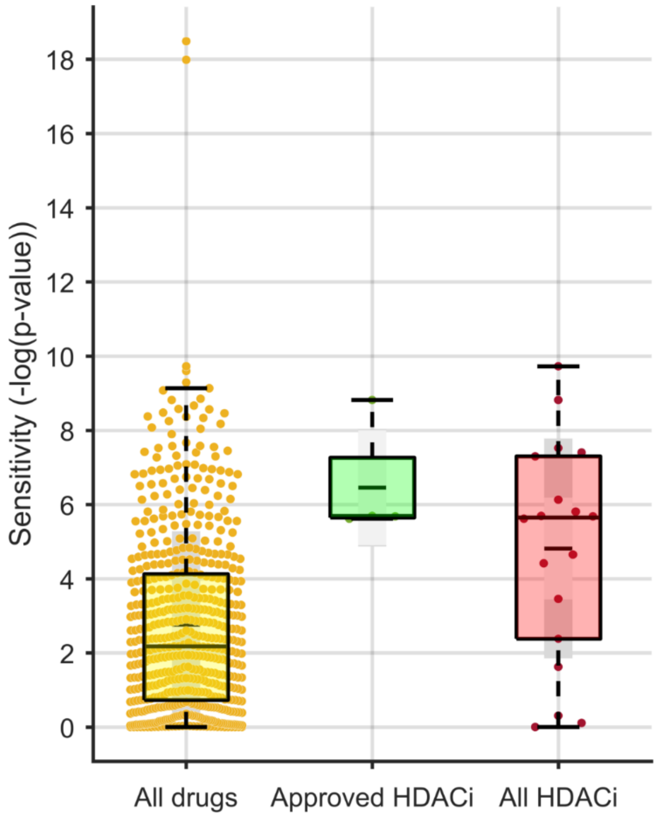

Fig. S4: Comparison of sensitivity between KDAC inhibitors and other drug classes in cell lines with high acetylation flux. We found that KDAC (or HDAC) inhibitors were significantly more sensitive against the high acetylation flux group than the low acetylation flux group of cell lines (p-value < 10^{-5}, t-test, Figure 5). The box plots show the relative sensitivity of all 546 compounds in the Seashore-Ludlow et al dataset between the high and low acetylation cell line group, quantified by the t-test p-value [1]. The average sensitivity of the clinically-used HDAC inhibitors – Vorinostat, Panobinostat, and Belinostat (FDA approved), and Entinostat (in clinical trial), was significantly higher in the high acetylation group than other drug classes (t-test p-value = 0.003). Similarly, the average sensitivity of all compounds that inhibit HDAC activity (red box) in the Seashore-Ludlow et al dataset was significantly higher in the high acetylation group than other drug classes (t-test p-value = 7 x 10^{-4}).

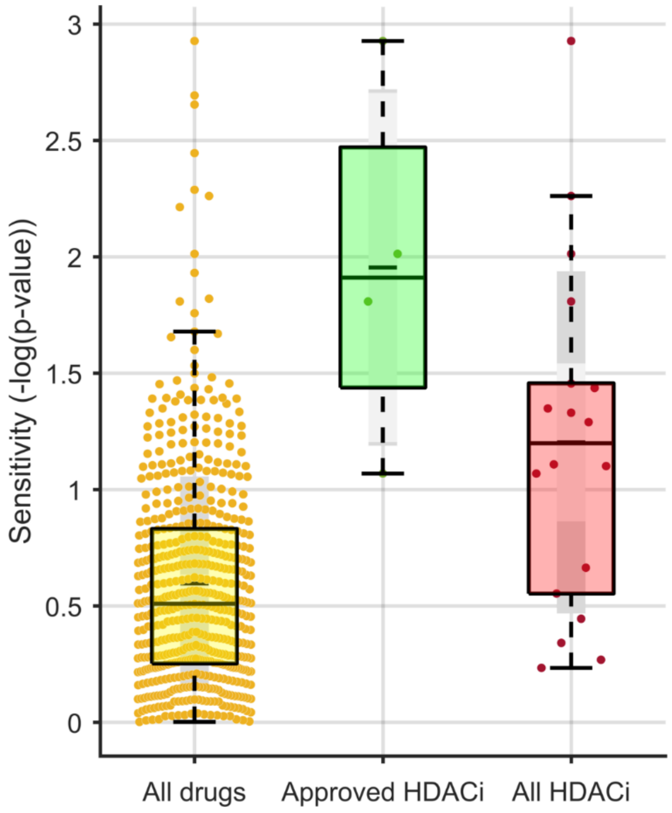

Fig. S5: Comparison of growth inhibition by KDAC inhibitors and other drugs in cell lines with high acetylation flux after controlling for doubling time. The box plots show the relative sensitivity of all compounds in the Seashore-Ludlow et al dataset between the high and low acetylation cell line group, quantified by the t-test p-value. We used sensitivity values from Hafner et al that corrected the sensitivity values in the Seashore-Ludlow et al dataset for the effect of the growth rate of the cell lines [2]. The average sensitivity of the HDAC inhibitors – Vorinostat, Panobinostat, Belinostat and Entinostat (green box), and all compounds that inhibit HDAC activity (red box) in the Seashore-Ludlow et al dataset was significantly higher in the high acetylation group than other drug classes (t-test p-value = 7 x 10^{-9} and 1 x 10^{-7} respectively)

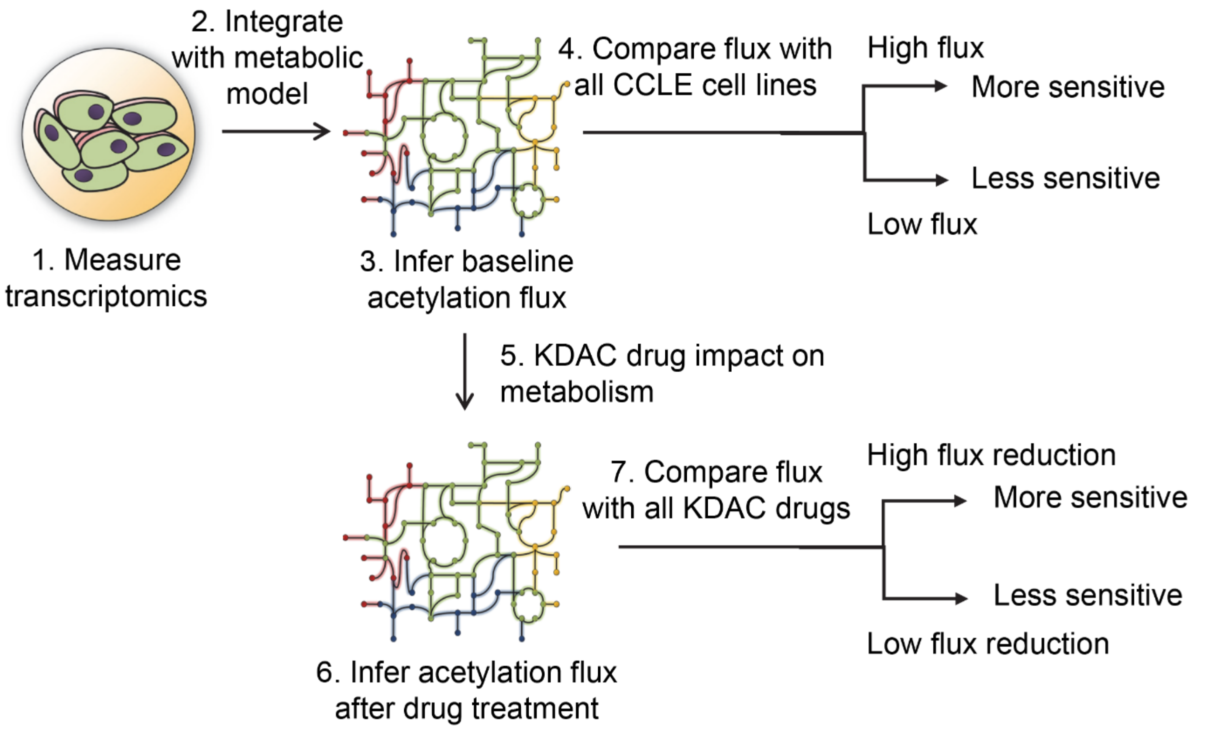

Fig. S6: Overview of the steps to predict the sensitivity of a new cell line to KDAC inhibitors using baseline (pre-treatment) transcriptomics data. The baseline acetylation flux can be used to predict if if the corresponding cell line will be more sensitive (high flux) or less sensitive (low flux) to all KDAC inhibitors on average (outlined in Figure 5). By further incorporating the impact of each KDAC inhibitor on the activity of metabolic enzymes, we can predict the relative sensitivity to each inhibitor based on the reduction in acetylation flux by each drug (outlined in Figure 6).

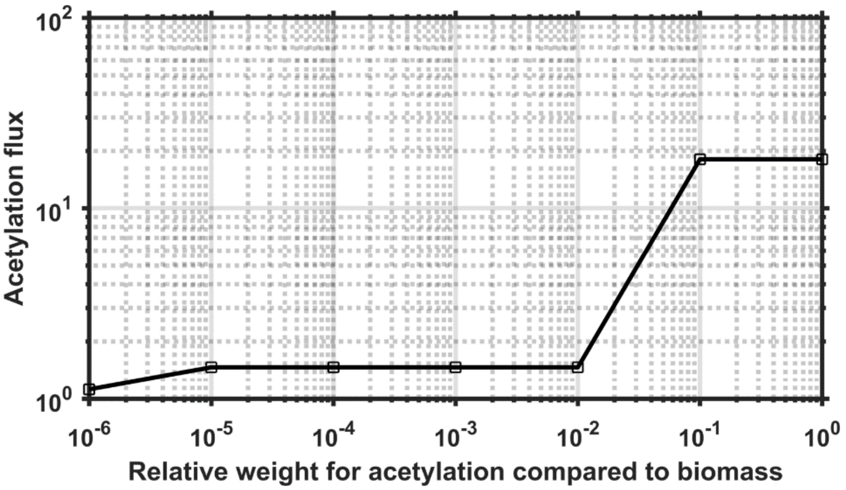

Fig. S7: Sensitivity analysis of the relative optimization weight for acetylation compared to biomass production. Choosing any small value (epsilon) between 0.01 to 10^{-6} produced the same value of acetylation flux (mmol/gDW cells/hr). Acetylation is assumed to be a secondary objective – it is optimized after biomass synthesis objective is maximized.

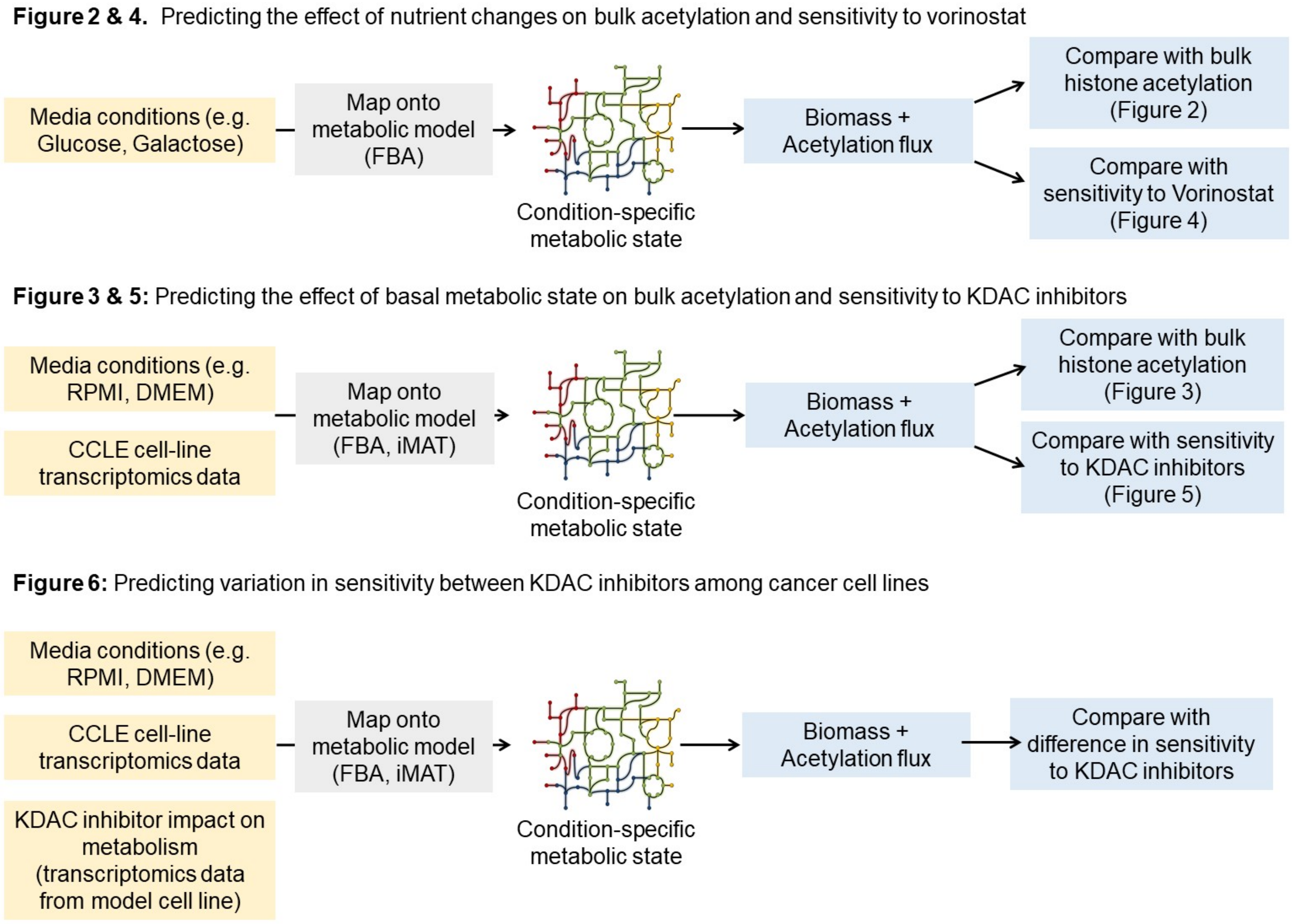

Fig. S8: Schematic overview of the inputs and outputs of various analyses performed in this study. The steps used to determine the impact of nutrients and transcriptome state on histone acetylation and sensitivity to KDAC inhibitors is shown for each analysis.

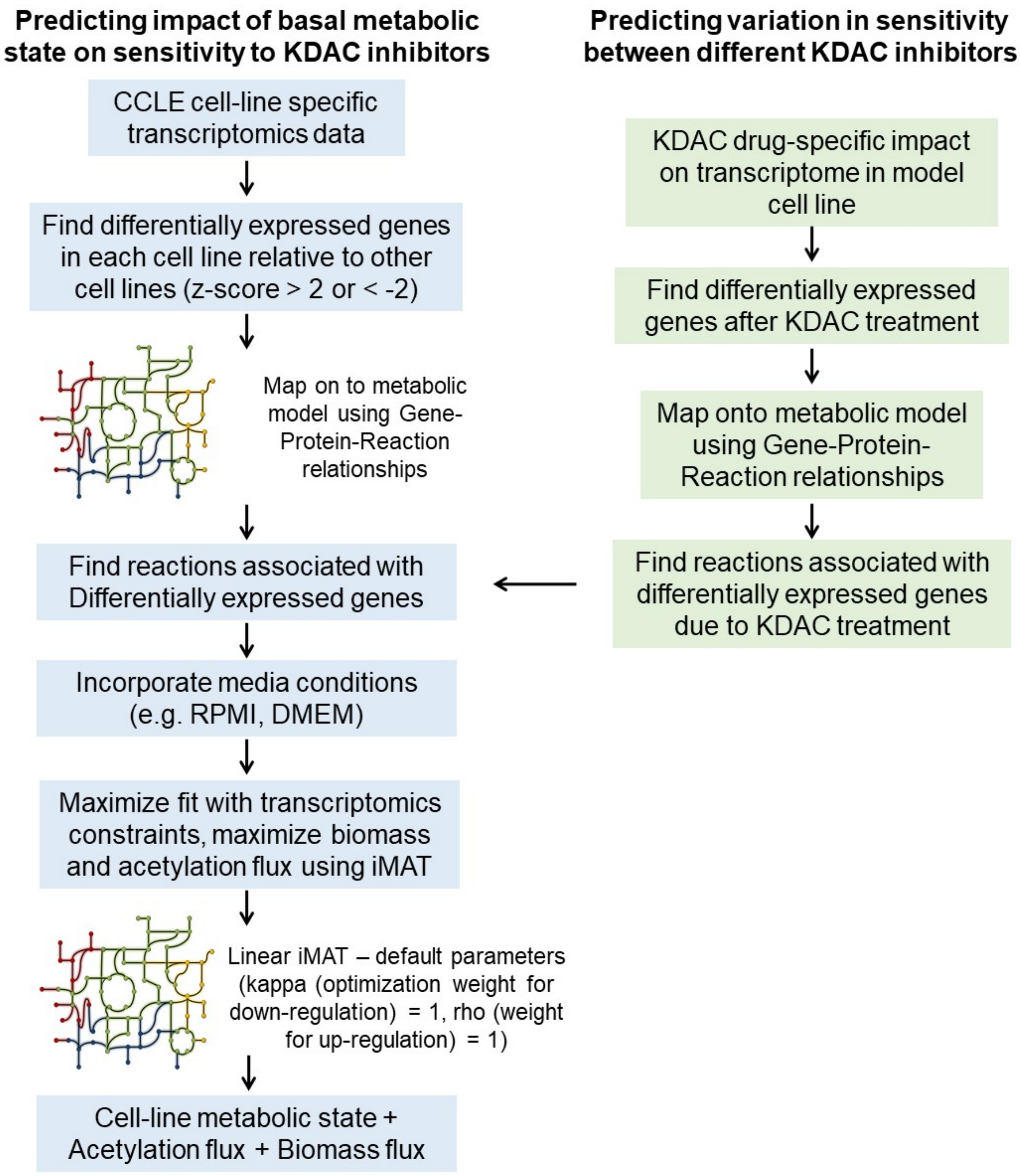

Fig. S9: The flowchart describes the steps for predicting acetylation flux in various cell lines and corresponding sensitivity to KDAC inhibitors using transcriptomics data. The transcriptomic data of specific KDAC drug treatment can be further overlaid onto the model to infer drug specific sensitivities.

Supplementary Tables

	Acetylation-enhancing metabolic genes	1. Acetylated?	2. Interacts with acetylase?	3. Interacts with deacetylase?
MTHFR	methylenetetrahydrofolate reductase (NAD(P)H)			
MTR	5-methyltetrahydrofolate-homocysteine methyltransferase			
DLD	dihydrolipoamide dehydrogenase	X	X	X
GCDH	glutaryl-CoA dehydrogenase			
SLC25A21	solute carrier family 25 (mitochondrial oxodicarboxylate carrier), member 21			
DLST	dihydrolipoamide S-succinyltransferase (E2 component of 2-oxo-glutarate complex)	X	X	X
OGDH	oxoglutarate (alpha-ketoglutarate) dehydrogenase (lipoamide)	X		X
PDHX	pyruvate dehydrogenase complex, component X	X	X	X
ACMSD	aminocarboxymuconate semialdehyde decarboxylase			
HAAO	3-hydroxyanthranilate 3,4-dioxygenase		X	
KMO	kynurenine 3-monooxygenase (kynurenine 3-hydroxylase)			
KYNU	kynureninase (L-kynurenine hydrolase)		X	X
TDO2	tryptophan 2,3-dioxygenase			X
BCKDHA	branched chain keto acid dehydrogenase E1, alpha polypeptide	X		X
BCKDHB	branched chain keto acid dehydrogenase E1, beta polypeptide	X		X
DBT	dihydrolipoamide branched chain transacylase E2	X		X
ASRGL1	asparaginase like 1			
AADAT	aminoadipate aminotransferase	X		
MUT	methylmalonyl CoA mutase	X		
CBS	cystathionine-beta-synthase		X	
CTH	cystathionase (cystathionine gamma-lyase)		X	X
CDO1	cysteine dioxygenase, type I		X	
FTCD	formiminotransferase cyclodeaminase			
HAL	histidine ammonia-lyase	X		
UROC1	urocanase domain containing 1			
SDS	serine dehydratase			
PCCA	propionyl CoA carboxylase, alpha polypeptide	X		
PCCB	Propionyl Coenzyme A carboxylase, beta polypeptide	X		X
MCEE	methylmalonyl CoA epimerase	X		
ACAT1	acetyl-CoA acetyltransferase 1	X	X	X
HSD17B10	hydroxysteroid (17-beta) dehydrogenase 10	X	X	X
PCBD1	pterin-4 alpha-carbinolamine dehydratase/dimerization cofactor of hepatocyte nuclear factor 1 alpha	X	X	X
PAH	phenylalanine hydroxylase			
HIBADH	3-hydroxyisobutyrate dehydrogenase		X	

AUH	AU RNA binding protein/enoyl-CoA hydratase			
IVD	isovaleryl-CoA dehydrogenase	X		
MCCC1	methylcrotonoyl-CoA carboxylase 1 (alpha)	X		
MCCC2	methylcrotonoyl-CoA carboxylase 2 (beta)			X
PRODH 2	proline dehydrogenase (oxidase) 2			

Table S1: List of 39 genes that increase acetylation when deleted in the model. Column 1 shows acetylation status based on protein acetylation database. Column 2 shows proteins that are found to physically interact with an acetylase and Column 3 shows proteins that are found to physically interact with a deacetylase enzyme (X mark implies true).

Acetylase/Deacetylase enzyme		Acetylation impacting metabolic enzyme	
HDAC5	histone deacetylase 5	CS	citrate synthase
HDAC5	histone deacetylase 5	OAT	ornithine aminotransferase
HDAC5	histone deacetylase 5	SLC25A1	solute carrier family 25 (mitochondrial carrier; citrate transporter), member 1
HDAC5	histone deacetylase 5	ACLY	ATP citrate lyase
SIRT7	sirtuin 7	ACLY	ATP citrate lyase
SIRT7	sirtuin 7	SDHA	succinate dehydrogenase complex, subunit A, flavoprotein (Fp)
SIRT7	sirtuin 7	OAT	ornithine aminotransferase
SIRT7	sirtuin 7	FH	fumarate hydratase
TERF2IP	telomeric repeat binding factor 2, interacting protein	TALDO1	transaldolase 1
RBM14	RNA binding motif protein 14	SDHB	succinate dehydrogenase complex, subunit B, iron sulfur (Ip)
IWS1	IWS1 homolog	SDHB	succinate dehydrogenase complex, subunit B, iron sulfur (Ip)
SIN3A	SIN3 homolog A, transcription regulator (yeast)	ACO2	aconitase 2, mitochondrial
PARK7	Parkinson disease (autosomal recessive, early onset) 7	TALDO1	transaldolase 1
ATF2	activating transcription factor 2	CS	citrate synthase
ATF2	activating transcription factor 2	OAT	ornithine aminotransferase
ATF2	activating transcription factor 2	FH	fumarate hydratase
HDAC1	histone deacetylase 1	IDH2	isocitrate dehydrogenase 2 (NADP+), mitochondrial
KAT2B	K(lysine) acetyltransferase 2B	ACLY	ATP citrate lyase
SIRT2	sirtuin 2	ACLY	ATP citrate lyase
SIRT6	sirtuin 6	ACLY	ATP citrate lyase
PPT1	palmitoyl-protein thioesterase 1	SLC25A1	solute carrier family 25 (mitochondrial carrier; citrate transporter), member 1
SMAD4	SMAD family member 4	SHMT1	serine hydroxymethyltransferase 1 (soluble)
SIRT3	sirtuin 3	SDHA	succinate dehydrogenase complex, subunit A, flavoprotein (Fp)
SIRT3	sirtuin 3	SDHB	succinate dehydrogenase complex, subunit B, iron sulfur (Ip)
WBP2	WW domain binding protein 2	SDHA	succinate dehydrogenase complex, subunit A, flavoprotein (Fp)
PARK7	Parkinson disease (autosomal recessive, early onset) 7	OAT	ornithine aminotransferase
RUVBL2	RuvB-like 2	SDHA	succinate dehydrogenase complex, subunit A, flavoprotein (Fp)
BRCA1	breast cancer 1, early onset	ACLY	ATP citrate lyase
BRCA1	breast cancer 1, early onset	TALDO1	transaldolase 1
ATXN3	ataxin 3	SDHB	succinate dehydrogenase complex, subunit B, iron sulfur (Ip)
ATXN3	ataxin 3	ACLY	ATP citrate lyase
ATXN3	ataxin 3	SDHA	succinate dehydrogenase complex, subunit A, flavoprotein (Fp)
ATXN3	ataxin 3	SLC25A1	solute carrier family 25 (mitochondrial carrier; citrate transporter), member 1
ATXN3	ataxin 3	SDHB	succinate dehydrogenase complex, subunit B, iron sulfur (Ip)

Table S2: List of physical interactions from the Biogrid database between metabolic enzymes predicted by the model to impact acetylation when deleted and acetylase or deacetylase enzymes.

Acetylase/Deacetylase enzyme		Acetylation enhancing metabolic enzyme	
ACTL6A	actin-like 6A	DLST	dihydrolipoamide S-succinyltransferase (E2 component of 2-oxo-glutarate complex)
ATF2	activating transcription factor 2	DLD	dihydrolipoamide dehydrogenase
BRCA1	breast cancer 1, early onset	HIBADH	3-hydroxyisobutyrate dehydrogenase
BRD7	bromodomain containing 7	PCBD1	pterin-4 alpha-carbinolamine dehydratase/dimerization cofactor of hepatocyte nuclear factor 1 alpha
BRD7	bromodomain containing 7	DLST	dihydrolipoamide S-succinyltransferase (E2 component of 2-oxo-glutarate complex)
CAMK1	Calcium/calmodulin-dependent protein kinase I	CDO1	cysteine dioxygenase, type I
CCAR2	CCAR2	HSD17B10	hydroxysteroid (17-beta) dehydrogenase 10
CHD3	chromodomain helicase DNA binding protein 3	HSD17B10	hydroxysteroid (17-beta) dehydrogenase 10
CTBP1	C-terminal binding protein 1	CTH	cystathionase (cystathionine gamma-lyase)
EP300	E1A binding protein p300	PDHX	pyruvate dehydrogenase complex, component X
HDAC1	histone deacetylase 1	HSD17B10	hydroxysteroid (17-beta) dehydrogenase 10
HDAC11	histone deacetylase 11	MCCC2	methylcrotonoyl-CoA carboxylase 2 (beta)
HDAC5	histone deacetylase 5	HSD17B10	hydroxysteroid (17-beta) dehydrogenase 10
HDAC5	histone deacetylase 5	ACAT1	acetyl-CoA acetyltransferase 1
HDAC6	histone deacetylase 6	BCKDHA	branched chain keto acid dehydrogenase E1, alpha polypeptide
HDAC6	histone deacetylase 6	BCKDHB	branched chain keto acid dehydrogenase E1, beta polypeptide
HDAC6	histone deacetylase 6	DBT	dihydrolipoamide branched chain transacylase E2
HDAC6	histone deacetylase 6	DLD	dihydrolipoamide dehydrogenase
HDAC6	histone deacetylase 6	DLST	dihydrolipoamide S-succinyltransferase (E2 component of 2-oxo-glutarate complex)
HDAC6	histone deacetylase 6	OGDH	oxoglutarate (alpha-ketoglutarate) dehydrogenase (lipoamide)
HDAC6	histone deacetylase 6	PDHX	pyruvate dehydrogenase complex, component X
HNF1A	HNF1 homeobox A	PCBD1	pterin-4 alpha-carbinolamine dehydratase/dimerization cofactor of hepatocyte nuclear factor 1 alpha
KDM1A	Lysine (K)-specific demethylase 1A	TDO2	tryptophan 2,3-dioxygenase
LYPLA1	lysophospholipase I	PCCB	Propionyl Coenzyme A carboxylase, beta polypeptide
MTA1	metastasis associated 1	DLST	dihydrolipoamide S-succinyltransferase (E2 component of 2-oxo-glutarate complex)
MTA2	metastasis associated 1 family, member 2	DLST	dihydrolipoamide S-succinyltransferase (E2 component of 2-oxo-glutarate complex)
NAA10	N(alpha)-acetyltransferase 10, NatA catalytic subunit	DLD	dihydrolipoamide dehydrogenase
NAA50	N(alpha)-acetyltransferase 50, NatE catalytic subunit	DLD	dihydrolipoamide dehydrogenase

NAA50	N(alpha)-acetyltransferase 50, NatE catalytic subunit	HSD17B10	hydroxysteroid (17-beta) dehydrogenase 10
PARK7	Parkinson disease (autosomal recessive, early onset) 7	HSD17B10	hydroxysteroid (17-beta) dehydrogenase 10
PARK7	Parkinson disease (autosomal recessive, early onset) 7	DLD	dihydrolipoamide dehydrogenase
PHB	prohibitin	HSD17B10	hydroxysteroid (17-beta) dehydrogenase 10
PPT1	palmitoyl-protein thioesterase 1	DBT	dihydrolipoamide branched chain transacylase E2
RBBP4	retinoblastoma binding protein 4	DLD	dihydrolipoamide dehydrogenase
RBBP7	retinoblastoma binding protein 7	DLD	dihydrolipoamide dehydrogenase
RBBP7	retinoblastoma binding protein 7	HSD17B10	hydroxysteroid (17-beta) dehydrogenase 10
RUVBL1	RuvB-like 1	HSD17B10	hydroxysteroid (17-beta) dehydrogenase 10
RUVBL1	RuvB-like 1	DLD	dihydrolipoamide dehydrogenase
RUVBL1	RuvB-like 1	HSD17B10	hydroxysteroid (17-beta) dehydrogenase 10
RUVBL2	RuvB-like 2	DLD	dihydrolipoamide dehydrogenase
RUVBL2	RuvB-like 2	HSD17B10	hydroxysteroid (17-beta) dehydrogenase 10
SAP18	Sin3A-associated protein, 18kDa	DLST	dihydrolipoamide S-succinyltransferase (E2 component of 2-oxo-glutarate complex)
SAP30	Sin3A-associated protein, 30kDa	PCBD1	pterin-4 alpha-carbinolamine dehydratase/dimerization cofactor of hepatocyte nuclear factor 1 alpha
SET	SET nuclear oncogene	DLD	dihydrolipoamide dehydrogenase
SFPQ	splicing factor proline/glutamine-rich	ACAT1	acetyl-CoA acetyltransferase 1
SIRT1	sirtuin 1	KYNU	kynureninase (L-kynurenine hydrolase)
SIRT7	sirtuin 7	ACAT1	acetyl-CoA acetyltransferase 1
SIRT7	sirtuin 7	DLD	dihydrolipoamide dehydrogenase
SMARC B1	SWI/SNF related, matrix associated, actin dependent regulator of chromatin, subfamily b, member 1	DLST	dihydrolipoamide S-succinyltransferase (E2 component of 2-oxo-glutarate complex)
TAF5	TAF5 RNA polymerase II, TATA box binding protein (TBP)-associated factor, 100kDa	HSD17B10	hydroxysteroid (17-beta) dehydrogenase 10
TERF2IP	telomeric repeat binding factor 2, interacting protein	HAAO	3-hydroxyanthranilate 3,4-dioxygenase
TP53	tumor protein p53	DLST	dihydrolipoamide S-succinyltransferase (E2 component of 2-oxo-glutarate complex)
ZNF451	zinc finger protein 451	PCBD1	pterin-4 alpha-carbinolamine dehydratase/dimerization cofactor of hepatocyte nuclear factor 1 alpha

Table S3: List of physical interactions from the Biogrid database between acetylase or deacetylase enzymes and metabolic enzymes predicted by the model to enhance acetylation when deleted.

Plate ID	Substrate	DMSO	Vorinostat	Ratio (Vorinostat/DMSO)	In Recon 1 model?
A01	NegativeControl	0.73	1.10	1.51	
A02	NegativeControl	1.04	1.48	1.42	
A03	NegativeControl	0.74	0.91	1.23	
A04	a-Cyclodextrin	0.72	0.98	1.37	
A05	Dextrin	13.14	11.96	0.91	
A06	Glycogen	14.28	13.96	0.98	X
A07	Maltitol	0.93	1.55	1.66	
A08	Maltotriose	11.24	9.10	0.81	X
A09	Maltose	14.76	12.42	0.84	X
A10	D-Trehalose	1.38	3.55	2.58	X
A11	D-Cellobiose	1.14	1.31	1.15	
A12	Gentiobiose	0.75	0.96	1.28	
B01	D-Glucose-6-Phosphate	7.62	7.76	1.02	
B02	D-Glucose-1-Phosphate	15.58	17.64	1.13	
B03	L-Glucose	0.80	1.18	1.47	
B04	D-(+)-Glucose	28.33	24.89	0.88	X
B05	D-(+)-Glucose	13.32	11.16	0.84	X
B06	D-(+)-Glucose	24.07	20.57	0.85	X
B07	3-MethylGlucose	0.75	1.02	1.35	
B08	a-Methyl-D-Glucoside	1.24	2.25	1.82	
B09	b-Methyl-D-Glucoside	1.19	1.63	1.37	
B10	Salicin	2.19	3.62	1.65	
B11	D-Sorbitol	2.37	4.53	1.91	
B12	N-Acetyl-D-Glucosamine	1.10	1.53	1.39	X
C01	D-GlucosaminicAcid	1.84	2.44	1.32	X
C02	D-GlucuronicAcid	0.63	0.92	1.46	
C03	ChondroitinSulfateC	0.68	1.07	1.58	
C04	Mannan	0.65	0.61	0.93	
C05	D-Mannose	14.78	12.57	0.85	X
C06	a-Methyl-D-Mannoside	0.60	1.21	2.02	
C07	D-Mannitol	1.21	2.12	1.76	
C08	N-Acetyl-D-Mannosamine	0.84	1.17	1.39	
C09	D-Melezitose	0.67	0.92	1.38	
C10	Sucrose	0.73	1.07	1.46	X
C11	Palatinose	0.66	0.91	1.37	
C12	Turanose	1.25	3.29	2.63	
D01	D-Tagatose	0.77	0.93	1.21	X
D02	L-Sorbose	0.47	0.81	1.70	
D03	L-Rhamnose	1.23	2.04	1.66	
D04	L-Fucose	0.69	1.38	2.00	X
D05	D-Fucose	0.70	1.14	1.63	
D06	D-Fructose-6-Phosphate	9.66	9.31	0.96	
D07	D-Fructose	5.21	4.86	0.93	X
D08	Stachyose	0.83	1.38	1.66	

D09	D-Raffinose	1.32	1.97	1.50	
D10	Lactitol	0.88	1.21	1.39	
D11	Lactulose	0.98	1.48	1.52	
D12	a-D-Lactose	0.92	1.01	1.10	X
E01	MelibionicAcid	0.90	0.83	0.92	
E02	D-Melibiose	1.26	1.72	1.36	
E03	D-Galactose	9.23	10.31	1.12	X
E04	a-Methyl-D-Galactoside	1.29	2.20	1.70	
E05	b-Methyl-D-Galactoside	1.00	1.68	1.68	
E06	n-acetyl-neuraminicacid	1.03	2.07	2.01	
E07	Pectin	1.66	2.88	1.74	
E08	Sedoheptulosan	1.20	2.13	1.78	
E09	Thymidine	0.48	1.08	2.26	X
E10	Uridine	4.30	5.78	1.34	X
E11	Adenosine	13.49	11.65	0.86	X
E12	Inosine	10.97	9.88	0.90	X
F01	Adonitol	2.51	2.00	0.80	
F02	L-Arabinose	1.04	1.07	1.02	X
F03	D-Arabinose	1.21	1.46	1.21	
F04	b-Methyl-D-Xyloside	0.90	0.60	0.66	
F05	Xylitol	5.86	6.57	1.12	X
F06	m-Inositol	1.42	1.73	1.22	X
F07	i-Erythritol	0.70	0.76	1.08	
F08	1,2-Propanediol	1.24	1.59	1.28	
F09	2-Aminoethanol	0.74	0.49	0.66	
F10	D,L-a-GlycerolPhosphate	0.85	1.32	1.55	
F11	Glycerol	0.69	0.72	1.04	X
F12	CitricAcid	1.15	0.95	0.82	X
G01	TricarballylicAcid	1.11	1.27	1.14	
G02	L-LacticAcid(DL)	5.54	5.92	1.07	X
G03	MethylD-Lactate	0.94	1.40	1.49	
G04	MethylPyruvate	1.31	2.00	1.53	
G05	PyruvicAcid	8.88	8.24	0.93	X
G06	a-Keto-GlutaricAcid	2.12	3.59	1.69	X
G07	SuccinamicAcid	15.04	12.46	0.83	
G08	SuccinicAcid	2.35	4.28	1.83	X
G09	MonoMethylSuccinate	8.20	7.12	0.87	
G10	L-MalicAcid	1.44	2.35	1.63	
G11	D-MalicAcid	0.59	0.98	1.65	
G12	m-TartaricAcid	0.80	1.04	1.30	
H01	AcetoaceticAcid	3.12	4.42	1.42	X
H02	g-AminoButyricAcid	1.18	1.28	1.09	X
H03	a-Keto-ButyricAcid	2.29	2.29	1.00	
H04	D,L-a-Hydroxy-ButyricAcid	2.77	3.31	1.19	X
H05	b-Hydroxy-ButyricAcid	1.15	2.34	2.04	
H06	g-Hydroxy-ButyricAcid	0.72	0.86	1.20	

H07	ButyricAcid	1.52	1.75	1.15	X
H08	2,3-Butanediol	0.85	1.08	1.27	
H09	3-Hydroxy2-Butanone	0.80	1.08	1.35	
H10	PropionicAcid	0.60	0.65	1.08	X
H11	AceticAcid	0.67	0.70	1.06	X
H12	HexanoicAcid	0.81	2.14	2.66	

Table S4: Biolog phenotype microarray data for treatment with vorinostat and control (DMSO). The table shows growth inhibition of He-La cells by vorinostat and DMSO, quantified by area under the growth curve (AUC), across different nutrient conditions measured using Biolog phenotype microarrays. Average of 4 replicates shown. Among these substrates, those that were present in the Recon1 model and showed growth in control wells (AUC > 2) were used for analysis.

Top 10 over-represented reactions in the high acetylation flux group	Percentage of over representation	FDR p-value
phosphatidylinositol-3,4,5-trisphosphate 3-phosphatase	10%	2.62E-06
phosphatidylinositol-3,4,5-trisphosphate 3-phosphatase, nuclear	10%	1.31E-06
ADPribose transport	10%	1.00E-06
NAD nucleosidase,extracellular	10%	8.36E-07
NADP nucleosidase,extracellular	10%	7.16E-07
acetate-CoA ligase (AMP-forming)	9%	3.59E-05
acetyl-CoA synthetase	9%	3.31E-05
ethanolamine phosphotransferase	8%	0.000123
Propionyl-CoA carboxylase, mitochondrial	8%	0.001111
nicotinamide-nucleotide adenylyltransferase, mitochondrial	7%	0.001646

Top 10 over represented reactions in the low acetylation flux group	Percentage of over representation	FDR p-value
deoxyuridine kinase (ATP:Deoxyuridine), mitochondrial	10%	1.19E-06
thymidine kinase (ATP:thymidine)	10%	8.93E-07
adentylate kinase (GTP)	8%	4.86E-05
adenylate kinase (d form)	8%	4.32E-05
cytidylate kinase (CMP)(GTP)	8%	3.88E-05
cytidylate kinase (dCMP)(GTP)	8%	3.53E-05
FAD diphosphatase	7%	0.00233
Cytochrome P450 27	6%	0.002014
Vitamin D-25-hydroxylase (D2)	6%	0.001951
Vitamin D-25-hydroxylase (D3)	6%	0.001892

Table S5: Top 10 reactions that differ between the CCLE cell lines predicted to have high and low acetylation flux.

Metabolic genes down regulated by KDACs (FDR p-value < 0.01)			
Panobinostat	Vorinostat	Entinostat	Belinostat
ME3	ADA	AGPAT4	ADA
	ALDH1A2	AGPS	PDE10A
	ME3	ALDH1A2	
	NDUFB1	ATP6V0A2	
	PDE10A	B3GNT5	
	PTGS1	BCMO1	
	SLC6A8	CERK	
		CHKB	
		ENTPD4	
		EXT1	
		GALC	
		GLUD2	
		GNPDA2	
		GUCY1B3	
		HMGCLL1	
		HMGCS1	
		HS2ST1	
		HS3ST3B1	
		ME3	
		MMAA	
		NDUFB1	
		NT5C2	
		PDE10A	
		PDE3B	
		PDE7A	
		PGAP1	
		PIK3CA	
		PIK3R1	
		PIK3R3	
		PLCH1	
		PLCXD2	
		PPOX	
		PRDX2	
		PTGIS	
		SLC2A12	
		SLC6A8	
		SORD	
		ST3GAL3	
		ST6GALNAC5	
		TMLHE	
		ZADH2	

Metabolic genes up regulated by KDACs (FDR p-value < 0.01)			
Panobinostat	Vorinostat	Entinostat	Belinostat
ALDH1A3	ABAT	ABAT	CYP1B1
CYP1B1	ACSL1	ACADSB	GAD1
GAD1	ALDH1A3	ACAT1	GALNT3
HAS2	ASNS	ACOX1	PIPOX
HNMT	ASS1	ACSL1	PLA2G2A
NMNAT2	B3GNT2	ACSS1	PLA2G4A
PDE3A	B3GNT7	ADCY2	
PLA2G2A	CA12	AGPAT2	
PLA2G4A	CYP1B1	AK2	
SLC7A11	ENPP2	AK3	
	GAD1	ALDH1A3	
	GALNT3	ALDH1B1	
	HNMT	ALOX5	
	NANS	ASNS	
	NMNAT2	ASS1	
	PDE3A	ATP1B2	
	PFKP	ATP6V0A4	
	PGM1	ATP6V0D2	
	PLA2G2A	B3GNT7	
	PLA2G4A	BDH1	
	PLA2G7	BLVRB	
	PLCE1	C1GALT1	
	SAT1	CA12	
	SLC16A1	CBR1	
	SLC1A3	CBS	
	SLC25A12	CHKA	
	SLC2A3	CHST4	
	SLC43A1	CKB	
	SLC7A11	COMTD1	
		COQ3	
		CPT1A	
		CTH	
		DCXR	
		DEGS1	
		DNMT3B	
		ENO1	
		ENO2	
		ENTPD1	
		ETNK1	
		FUT1	
		GAD1	
		GALNT3	
		GALNT6	
		GART	

		GCLM	
		GLS2	
		GPX3	
		HAS3	
		HK1	
		HMOX1	
		HS3ST5	
		HSD11B2	
		IDH1	
		IDH3A	
		IDO1	
		LDHD	
		MAN1A1	
		MAN1C1	
		MGST2	
		MTHFD1	
		MTHFD2	
		NAGLU	
		NANS	
		NDUFB9	
		NMNAT2	
		NOS1	
		OAT	
		P4HA2	
		PC	
		PDE2A	
		PDE3A	
		PDE4A	
		PDE8A	
		PFKP	
		PGM1	
		PIGW	
		PIK3CB	
		PIPOX	
		PLA2G2A	
		PLA2G3	
		PLA2G4A	
		PLA2G7	
		PLOD2	
		PMM2	
		PPAP2A	
		PSAT1	
		SEPHS1	
		SGPP1	
		SHMT2	
		SLC13A5	

		SLC16A1	
		SLC16A3	
		SLC24A3	
		SLC25A12	
		SLC2A3	
		SLC38A5	
		SLC3A2	
		SLC43A1	
		SLC4A8	
		SLC7A1	
		SLC7A10	
		SLC7A11	
		SLC7A3	
		SLC7A5	
		SLCO4A1	
		SMS	
		SOAT1	
		SPTLC2	
		SULT1A1	
		SULT1A2	
		SULT2B1	
		TPI1	
		UGP2	

Table S6: List of genes that were up- or down-regulated by each KDAC inhibitor. This list of genes was overlaid uniformly onto all CCLE metabolic models to infer the impact of KDAC treatment on acetylation flux. Data from Rempel et al [3].

Analysis	10^{-2}	10^{-3}	10^{-4}
Acetylation-impacting genes (% similarity compared to the gene list at epsilon = 10^{-3}) (Figure 2)	100%	100%	100%
Correlation with bulk H3K9 histone acetylation (Figure 3)	R = 0.6	R = 0.6	R = 0.6
Correlation with sensitivity to vorinostat (Figure 4)	R = -0.67	R = -0.67	R = -0.67
Predicting CCLE cell line sensitivity to KDACs (Figure 5)<br><br>Difference in sensitivity between high acetylation flux and low acetylation flux cell line group, p-value t-test:	Panobinostat - 4.06 X 10^{-6}<br>Vorinostat - 4.18 X 10^{-6}<br>Belinostat - 3.05 X 10^{-9}<br>Entinostat - 4.87 X 10^{-6}	Panobinostat - 3.8 X 10^{-6}<br>Vorinostat - 4.6 X 10^{-6}<br>Belinostat - 4.0 X 10^{-9}<br>Entinostat - 5.8 X 10^{-6}	Panobinostat - 4.2 X 10^{-6}<br>Vorinostat - 4.7 X 10^{-6}<br>Belinostat - 3.0 X 10^{-9}<br>Entinostat - 4.87 X 10^{-6}
Predicting variation in sensitivity to KDACs among CCLE cell lines (Figure 6)<br><br>Difference in growth inhibition for cell lines with high acetylation flux reduction for a KDAC drug relative to others:	2.6 AUC units, p-value = 10^{-62}, t-test	2.67 AUC units, p-value = 10^{-67}, t-test	2.54 AUC units, p-value = 10^{-56}, t-test

Table S7: Robustness of our results to the choice of the relative optimization weight for acetylation compared to biomass synthesis. Our results were identical at three different optimization weights. The default weight used is 10^{-3}. As shown in Fig. S7, the acetylation flux was identical for a broad range of optimization weights from 0.01 to 10^{-5}.

**Analysis involving transcriptomics integration**	**iMAT**	**GIMME**
Correlation with bulk H3K9 histone acetylation (Figure 3)	R = 0.6	R = 0.59
Predicting CCLE cell line sensitivity to KDACs (Figure 5)<br><br>Difference in sensitivity between high acetylation flux and low acetylation flux cell line group, p-value t-test:	Panobinostat - 4.06 X 10^{-6}<br>Vorinostat - 4.18 X 10^{-6}<br>Belinostat - 3.05 X 10^{-9}<br>Entinostat - 4.87 X 10^{-6}	Panobinostat - 7.8 X 10^{-6}<br>Vorinostat – 2.6 X 10^{-6}<br>Belinostat - 3.0 X 10^{-9}<br>Entinostat - 4.3 X 10^{-6}
Predicting variation in sensitivity to KDACs among CCLE cell lines (Figure 6)<br><br>Difference in growth inhibition for cell lines with high acetylation flux reduction for a KDAC drug relative to others:	2.6 AUC units, p-value = 10^{-62}, t-test	1.65 AUC units, p-value = 10^{-31}, t-test

Table S8: Robustness of our results to the choice of the method for transcriptomics integration. In addition to the iMAT approach, we repeated all our analyses with an alternative approach for transcriptomics data integration called GIMME. GIMME assumes a linear association between extent of downregulation of a gene's expression and the downregulation of flux through the corresponding enzyme encoded by it. We found that our results from this approach are consistent with our results using iMAT.

References

1. Seashore-Ludlow B, Rees MG, Cheah JH, Coko M, Price E V., Coletti ME, et al. Harnessing connectivity in a large-scale small-molecule sensitivity dataset. Cancer Discov. 2015;5:1210–23.

2. Hafner M, Niepel M, Chung M, Sorger PK. Growth rate inhibition metrics correct for confounders in measuring sensitivity to cancer drugs. Nat Methods. 2016;13:521–7.

3. Rempel E, Hoelting L, Waldmann T, Balmer N V., Schildknecht S, Grinberg M, et al. A transcriptome-based classifier to identify developmental toxicants by stem cell testing: design, validation and optimization for histone deacetylase inhibitors. Arch Toxicol. 2015;89:1599–618.
